# Per- and Polyfluoroalkyl Substances Induce Cardiotoxicity and Alter Protein Profiles of Extracellular Matrix, Metabolism, and Mitochondrial Function in Human Cardiomyocytes

**DOI:** 10.1021/acs.chemrestox.5c00267

**Published:** 2025-11-15

**Authors:** Wenhao Zhang, Zeyu Wang, Olivia Reid, Frank Harris, Kun Man, Matthew Wang, Stephanie Li, Lawrence C. Armand, Alicia Lane, Gayatri Patel, Victor Faundez, Yuhong Du, Ronghu Wu, Lou Ann Brown, W. Michael Caudle, Chunhui Xu

**Affiliations:** † Department of Pediatrics, 1371 Emory University School of Medicine and Children’s Healthcare of Atlanta, Atlanta, Georgia 30322, United States; ‡ School of Chemistry and Biochemistry and the Petit Institute for Bioengineering and Bioscience, 1372 Georgia Institute of Technology, Atlanta, Georgia 30332, United States; § Department of Cell Biology, 12239 Emory University School of Medicine, Atlanta, Georgia 30322, United States; ∥ Wallace H. Coulter Department of Biomedical Engineering, Georgia Institute of Technology and Emory University, Atlanta, Georgia 30332, United States; ⊥ Department of Pharmacology and Chemical Biology, Emory University School of Medicine, Atlanta, Georgia 30322, United States; # Gangarosa Department of Environmental Health, Rollins School of Public Health, Emory University, Atlanta, Georgia 30322, United States

## Abstract

Per- and polyfluoroalkyl substances (PFAS), common environmental contaminants, can cause cardiotoxic effects particularly during fetal development. However, the effect of combined PFAS exposure, which more closely reflects real-world environmental conditions, remains poorly understood. In this study, human induced pluripotent stem cell–derived cardiomyocytes (hiPSC-CMs) were exposed to three common PFAS compounds—perfluorohexanesulfonic acid (PFHxS), perfluorooctanoic acid (PFOA), and perfluorodecanoic acid (PFDA)—individually or in combination (20–200 μM; consistent with serum levels reported in occupationally exposed populations). Compared with single compounds, combined PFAS exposure induced synergistic cytotoxicity, significantly reducing hiPSC-CM viability after 5 or 10 days. Sublethal combined exposure for 10 days altered mitochondrial membrane potential and mitochondrial content in a dose-dependent manner and shifted cysteine metabolism, potentially reflecting adaptation to oxidative challenge. After 14 days, combined PFAS increased vimentin, a fibroblast marker, and reduced NKX2.5, α-actinin, and cardiac troponin T, key markers of cardiomyocytes, as detected by immunocytochemistry. Proteomics further showed enrichment of pathways in extracellular matrix organization, cholesterol metabolism, and antioxidant defense, as well as downregulation of mitochondrial proteins. Consistent with changes in protein profiles related to oxidative stress and bioenergetic impairment, exposure of hiPSC-CMs to combined PFAS also increased the level of mitochondrial superoxide, reduced ATP content, and decreased cellular respiration. Together, these data demonstrate that PFAS mixtures drive mitochondrial dysfunction, oxidative stress, metabolic changes, and extracellular matrix remodeling in hiPSC-CMs, underscoring the importance of evaluating PFAS mixtures to better understand cardiac risks from environmental exposure.

## Graphical abstract

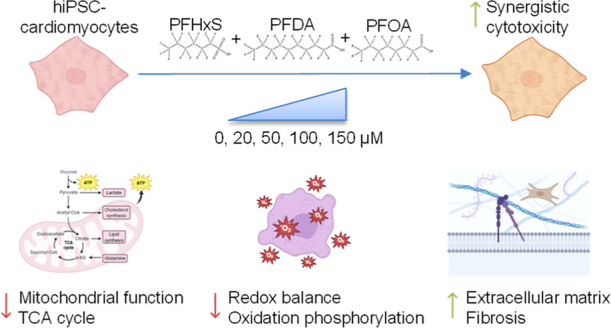

## Introduction

Per- and polyfluoroalkyl substances (PFAS) are a diverse group of synthetic organic compounds characterized by fully or partially fluorinated carbon chains, which confer exceptional chemical stability and resistance to water, stains, and grease.^1,2^ These properties have led to their extensive use in industrial and consumer products, including nonstick cookware, stain-resistant fabrics, food packaging, firefighting foams, and water-repellent coatings.^2–4^ However, their widespread application and extreme environmental persistence have raised serious health concerns, as PFAS have been detected in drinking water, soil, wildlife, and human biological samples, including serum and breast milk.^5,6^ Notably, perfluorohexanesulfonic acid (PFHxS) concentrations as high as 219 μM have been reported in the serum of occupationally exposed populations.^7^ Due to their persistence in the environment and potential health risks, the U.S. Environmental Protection Agency (EPA) has classified PFAS exposure as an urgent public health concern.

An increasing number of studies have highlighted the potential cardiovascular effects of PFAS exposure. Epidemiological research has shown a positive association between PFAS exposure and cardiovascular diseases and peripheral arterial diseases.^8^ Emerging evidence from both epidemiological and *in vitro* studies suggests that PFAS contribute to cardiovascular dysfunction. For example, a study analyzed PFAS concentrations in maternal plasma and assessed cardiometabolic risk scores in children. The findings revealed that prenatal exposure to PFHxS was significantly associated with an increased cardiometabolic risk score.^9^ This is particularly concerning given that PFAS can cross the placenta and accumulate in various fetal tissues, including the heart,^5^ raising the possibility of direct developmental cardiotoxicity. Similarly, another study found that prenatal exposure to perfluorooctanoic acid (PFOA) and PFHxS was strongly correlated with increased cardiometabolic risk scores.^10^ Moreover, infants exposed to higher levels of perfluorodecanoic acid (PFDA) had a 2.33-fold increased risk of developing septal defects compared with those exposed to lower levels.^11^

Despite the growing epidemiological evidence linking PFAS to cardiovascular toxicity, the underlying mechanisms remain incompletely understood. To address this gap, human-induced pluripotent stem cell-derived cardiomyocytes (hiPSC-CMs) have emerged as a promising model for studying disease phenotypes, drug screening, and regenerative approaches.^12^ hiPSC-CMs provide a human-relevant platform to investigate the molecular mechanisms underlying environmental toxicant-induced cardiotoxicity. Recent studies suggest that PFAS exposure may induce cardiotoxicity through several mechanisms, including altered gene expression [NK2 Homeobox 5 (*NKX2-5*), Myosin Light Chain 4 (*MYL4*)],^13^ mitochondrial dysfunction,^14^ disruption of calcium homeostasis,^15^ and dysregulation of key signaling pathways such as Peroxisome Proliferator-Activated Receptor (PPAR)^16^ and Wnt.^13^

Previous studies have focused on the effects of individual PFAS compounds.^17^ However, this does not reflect real-world exposure scenarios, where humans are typically exposed to mixtures of multiple PFAS. While toxicity in HepG2 liver cells has been evaluated following exposure of the cells to PFAS mixtures,^18^ the effects of combined PFAS on cardiomyocytes remain largely unexplored.

Given the knowledge gap in combined PFAS exposure-induced cardiotoxicity, our study aims to assess the cytotoxic effects of PFOA, PFDA, and PFHxS, both individually and in combination on hiPSC-CMs. These compounds are commonly used in industry and detected in products and human serum.^19–21^ Understanding these effects will provide insight into the potential mechanisms underlying PFAS-induced cardiac dysfunction, which is essential for informing public health policies and regulatory guidelines.

## Materials and Methods

### Cell Culture

hiPSC line iPS (IMR90)-4 cells (WiCell Research Institute) were cultured on Matrigel-coated plates and maintained in mTeSR medium (Stemcell Technologies) with daily feeding. Cells were induced to differentiate into cardiomyocytes. hiPSCs were cultured in RPMI medium with 2% B27 minus insulin (B27 minus insulin medium) containing 6 μM CHIR99021 (SelleckChem) for 48 h, followed by incubation in B27 minus insulin medium for an additional 24 h. On day 3, the medium was replaced with 2 mL of B27 minus insulin medium supplemented with 5 μM IWR-1 (Sigma- Aldrich) for 48 h. From day 5 onward, RPMI medium with 2% B27 (B27 medium) was refreshed every other day until day 10. The lactate selection using glucose-free RPMI medium supplemented with 2% B27 and 5 mM sodium l-lactate was conducted on day 10 for 72 h. On day 17, cells were dissociated and replated in Matrigel-coated 96-well plates at a density of 1.5 × 10^4^ cells per well. Cells were exposed to PFAS (PFOA, PFDA, and PFHxS individually or in combination) at various concentrations, with DMSO serving as the control. For combined PFAS, each PFAS was added at the indicated concentration (e.g., 100 μM each). The PFAS were obtained from Sigma-Aldrich and dissolved in DMSO. The cell culture reagents were obtained from Thermo Fisher Scientific unless otherwise specified.

### Cell Viability and Mitochondrial Membrane Potential

Cell viability was assessed after the PFAS exposure of the cells initially seeded at 1.5 × 10^4^ cells per well in 150 μL of medium in 96-well plates. CellTiter-Blue assay reagent (30 μL, Promega) was added to each well, and cells were incubated for 4 h. Fluorescence was measured at 560Ex/590Em using a BioTek Cytation 5 Cell Imaging Platform (Agilent Technologies). For mitochondrial membrane potential assay, cells were stained with 100 nM tetramethylrhodamine (TMRM, Thermo Fisher Scientific) and 7 μM Hoechst working solution in warm 0.25% bovine serum albumin (BSA) in PBS for 15 min at 37 °C in the dark. MitoTracker Red (Thermo Fisher Scientific) staining involved incubating cells with 50 nM solution in PBS for 45 min at 37 °C in the dark, followed by Hoechst staining. Mean fluorescence intensity (MFI) was recorded using the BioTek Cytation 5.

### Mitochondrial Redox Analysis

To quantify the reduced and oxidized forms of glutathione (GSH/GSSG) and cysteine/cystine (Cys/CySS) by HPLC, mitochondria were promptly isolated using a Percoll density gradient, as previously described.^22^ Briefly, immediately following isolation, mitochondrial samples were acidified with 5% (v/v) perchloric acid containing 5 mM γ-glutamylglutamate as an internal standard. After acidification, the mitochondrial samples were derivatized with iodoacetic acid and dansyl chloride. GSH, GSSG, Cys, and CySS were separated by HPLC using an amino μBondapak column and quantified with fluorescence detection (Waters, Milford, MA). Concentrations were determined relative to the internal standard and normalized to cell number.

### Immunocytochemistry and High-Content Imaging Quantification

Immunocytochemistry was performed after the PFAS exposure. Cells were fixed with 4% paraformaldehyde for 15 min, then permeabilized with ice-cold methanol for 2 min at room temperature. Blocking was done with 5% normal goat serum (NGS) in PBS for 1 h at 4 °C on a shaker. Primary antibodies (Table S1, NKX2.5, cardiac troponin T (cTnT), α-actinin, and vimentin) were applied in 3% NGS overnight at 4 °C. After washing with PBS, cells were incubated with secondary antibodies (Table S1) at room temperature for 1 h in the dark and counterstained with Hoechst 33342. Images and cell counts were obtained using the BioTek Cytation 5, with cell numbers determined by Hoechst staining of nuclei. For quantification, a primary mask was used to restrict the detection of α-actinin, vimentin, and cTnT to 10 units from the nucleus. MFI and proportion of positive cells were used as outcome measurements.

### Proteomics Analysis

Proteomics analysis was performed after the PFAS exposure for 14 days to compare hiPSC-CM cultures exposed to 100 μM combined PFAS with those treated with DMSO control.^23^ Briefly, cells were lysed in a buffer containing 50 mM HEPES (pH 8.0), 150 mM NaCl, 0.5% sodium deoxycholate (SDC), 25 units/mL benzonase, and protease inhibitors (one tablet per 10 mL). The lysates were incubated at 4 °C for 45 min with end-overend rotation. Protein concentrations were measured using the bicinchoninic acid assay, and equal amounts of protein from each sample were used for subsequent analyses. Proteins were reduced with 5 mM dithiothreitol at 56 °C for 30 min and alkylated with 14 mM iodoacetamide at room temperature for 30 min in the dark. Subsequently, proteins were precipitated using the methanol–chloroform method and digested with trypsin. Peptides were purified using tC18 Sep-Pak cartridges. For running LC- MS/MS, Peptide samples were dissolved in 5% acetonitrile (ACN) and 4% formic acid (FA), then loaded onto a microcapillary column packed with C18 beads (Magic C18AQ, 1.9 μm, 200 Å, 75 μm × 16 cm). Peptides were eluted using a 95 min gradient of 2–24% ACN with 0.125% FA on a reverse-phase nano HPLC system (Dionex Ultimate 3000 RSLC Nano). An Orbitrap Exploris 480 mass spectrometer (Thermo Fisher Scientific) was employed for data-independent acquisition (DIA) analysis as described.^24,25^ MS1 spectra were collected over an *m*/*z* range of 420–680 at a resolution of 60,000, with an automatic gain control (AGC) target of 3 × 10^6^. MS2 spectra were acquired over an *m*/*z* range of 430–670 at a resolution of 30,000, using isolation windows of 4 *m*/*z* and stepped normalized collision energies of 22%, 26%, and 30%.

Raw files were processed against the *Homo sapiens* proteome database from UniProt (downloaded on 2023-03-06) using FragPipe (v21.1) with MSFragger (v4.0).^26^ The “DIA_SpecLib_Quant” workflow was utilized for spectral library construction and protein quantification.^27^ Parameters included a precursor and fragment mass tolerance of 20 ppm, fully tryptic enzyme specificity with a maximum of one missed cleavage, and peptide lengths between 7 and 50 amino acids. Variable modifications accounted for methionine oxidation (+15.9949 Da) and protein N-terminal acetylation (+42.0106 Da), while cysteine alkylation (+57.02146 Da) was set as a fixed modification. Peptide-spectrum matches (PSMs) and protein identifications were filtered to achieve a false discovery rate (FDR) of less than 1% using the Philosopher toolkit.^28^ Quantification was performed using the DIA-NN module with default settings. Statistical analysis and visualization were conducted using FragPipe-Analyst, employing variance stabilizing normalization and default parameters.^29^

Differentially expressed proteins were identified based on | log_2_(fold change) | ≥ 1 and *p*-value < 0.05. Functional annotation and pathway enrichment analyses, including Gene Ontology (GO) and Kyoto Encyclopedia of Genes and Genomes (KEGG) pathway analyses, were performed using the Database for Annotation, Visualization, and Integrated Discovery (DAVID). KEGG pathway Mapper analysis was performed using Pathview R package 1.48.0.

### Mitochondrial Oxidative Stress and ATP Content Assays

Mitochondrial oxidative stress was assessed using the MitoSOX Red assay (Thermo Fisher Scientific) as a measure of mitochondrial superoxide production. After the PFAS exposure, cells were incubated with MitoSOX Red and the nuclear stain Hoechst 33342 for 30 min at 37 °C in the dark. Fluorescent images were captured using a high-content imaging system, BioTek Cytation 5, and mitochondrial superoxide levels were quantified as the mean MitoSOX Red fluorescence intensity per cell. Intracellular ATP content was measured using the CellTiter-Glo 3D luminescent assay (Promega). After the cells were exposed to PFAS or DMSO for 14 days, plates were equilibrated to room temperature and an equal volume of CellTiter-Glo 3D reagent was added to each well, followed by gentle mixing and incubation for 30 min. Luminescence proportional to ATP content was then measured on the BioTek Cytation 5.

### Seahorse XF96 Cell Mito Stress Test

Cellular respiration was analyzed using the XF96 Cell Mito Stress Kit (Agilent Technologies). hiPSC-CMs were treated for 18 days with 100 μM combined PFAS or vehicle prior to metabolic assays. After the exposure, cells were dissociated using 0.25% trypsin/EDTA and plated into a poly-d-lysine-coated Seahorse XF96 plate at a density of 3 × 10^4^ cells/well with medium containing 10 μM ROCK inhibitor Y-27632. After 24 h, the cells were washed with base medium (XF RPMI medium + 5 mM glucose + 2 mM l-glutamine + 0.5 mM sodium pyruvate) and incubated in 180 μL base medium/well at 37 °C in a non-CO_2_ incubator. Oligomycin (2 μM), carbonyl cyanide p-(trifluoromethoxy) phenylhydrazone (FCCP, 1 μM) and rotenone (0.5 μM) + antimycin A (0.5 μM) were diluted in base medium and sequentially injected into each well during the measurements of oxygen consumption (OCR) and extracellular acidification rate (ECAR). The results were normalized to protein concentration using Pierce bicinchoninic acid Protein Assay Kit (Thermo Fisher Scientific) as described.^30^

### Statistical Analysis

Data were analyzed and graphed in GraphPad Prism 10 (GraphPad, San Diego, CA) and RStudio. Data are presented as mean ± standard deviation. Comparisons were conducted via Welch’s *t*-test or the one-way ANOVA test followed by Dunnett’s multiple comparison. Significant differences were defined by *p* < 0.05 (*), *p* < 0.01 (**), *p* < 0.001 (***), and *p* < 0.0001 (****). Statistical analysis for proteomics is described in the section of proteomics analysis.

## Results

### Combined PFAS Exposure Synergistically Reduces hiPSC-CM Viability

To evaluate the cytotoxic effects of PFAS, hiPSCs were differentiated into cardiomyocytes utilizing a Wnt signaling modulation approach^31,32^ (Figure 1a). On differentiation day 18, over 80% of the cells in cultures were positive for cardiac marker NKX2.5 (a cardiac transcription factor) as detected by high-content imaging analysis (Figure 1b).

**Figure 1:**
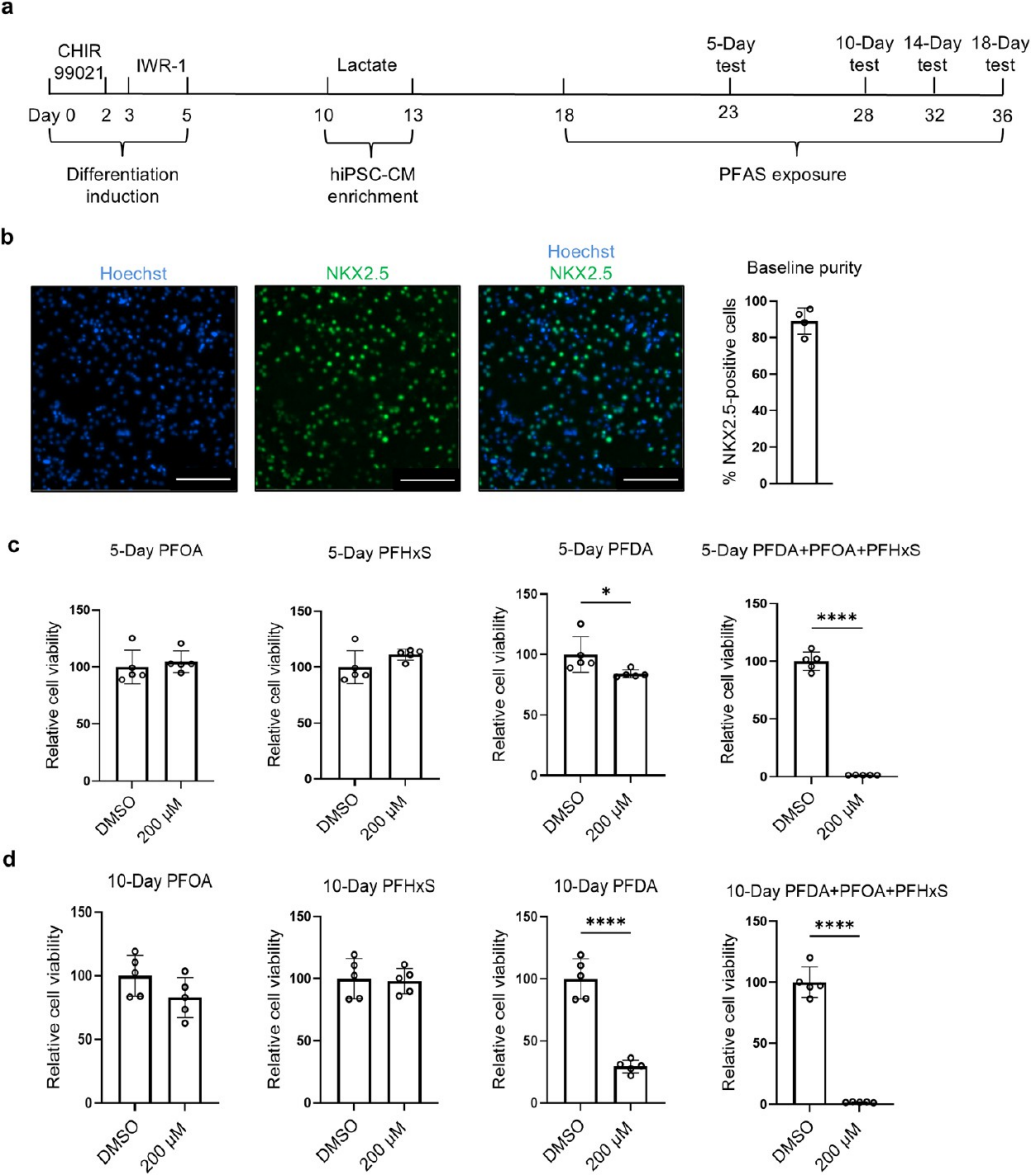
Combined PFAS exposure results in synergistic cytotoxicity on hiPSC-CMs. (a) Experimental design. (b) Representative images of NKX2.5 staining and quantification of proportion of NKX2.5-positive cells in hiPSC-CM cultures on differentiation day 18. Red: NKX2.5; blue: nuclei. (c) Cell viability was tested by CellTiter-Blue assay in hiPSC-CM cultures after the cells were treated with individual or combined PFAS of PFOA, PFDA, and PFHxS, for 5 days (c) and 10 days (d). Scale bars: 200 μm. Comparisons were conducted via Welch’s *t*-test. *p < 0.05; ****p < 0.0001. *n* = 4 cultures.

For cytotoxicity testing, hiPSC-CMs were treated with three specific PFAS compounds—PFOA, PFDA, and PFHxS—individually as well as in combination. Based on prior studies,^33–35^ the exposure concentration was set at 200 μM for each PFAS for the initial testing. After a 5-day exposure, only PFDA significantly reduced cell viability by 16% (*p* < 0.05), while PFOA and PFHxS showed no significant changes compared with cells treated with DMSO (control) (Figure 1c). Notably, the combined exposure to all three PFAS compounds resulted in a profound cytotoxic effect, reducing cell viability by 98.6% (*p* < 0.0001), suggesting synergistic interactions (Figure 1c). Further tests with 200 μM of combined PFAS for 10 days also indicated similar synergistic cytotoxic effects (Figure 1d). These results demonstrate that combined PFAS exposure significantly reduced the viability of hiPSC-CMs, suggesting enhanced cytotoxicity under mixed compound conditions.

In subsequent experiments, we focused on the sublethal effects of combined PFAS exposure on hiPSC-CMs. To determine an appropriate exposure concentration, hiPSC-CMs were treated with combined PFAS for 10 days at various concentrations (20 μM, 50 μM, 100 μM, and 150 μM), with DMSO as a negative control. As shown in Figure 2a, exposure to 150 μM PFAS for 10 days significantly decreased cell viability by 77.3% (*p* < 0.0001). These findings indicate that combined PFAS exposure significantly reduced hiPSC-CM viability in a dose-dependent manner.

**Figure 2:**
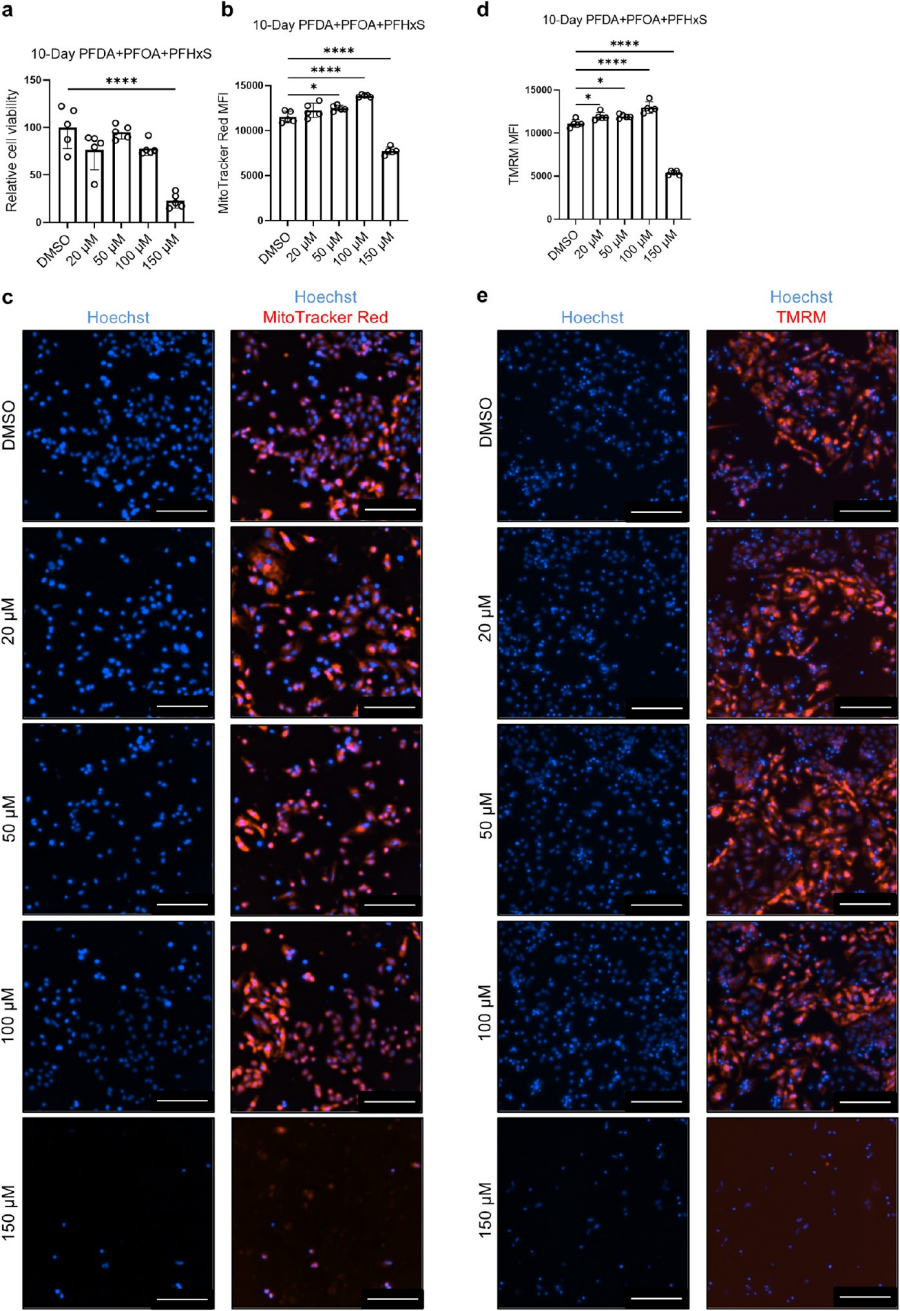
Combined PFAS exposure on hiPSC-CMs induces defects in mitochondrial features. hiPSC-CM cultures were treated with combined PFAS or DMSO for 10 days and then analyzed for cell viability and mitochondrial features. (a) Quantification of cell viability. (b) Quantification of MitoTracker Red mean fluorescence intensity (MFI). (c) Representative images of MitoTracker Red staining. (d) Quantification of TMRM MFI. (e) Representative images of TMRM staining. Scale bars: 200 μm. Comparisons were conducted via one-way ANOVA test followed by Tukey’s multiple comparisons test. *p < 0.05; ****p < 0.0001. *n* = 4 cultures.

### Combined PFAS Exposure Induces Mitochondrial Defects in hiPSC-CMs

To assess whether the PFAS exposure induced mitochondrial defects, mitochondrial features were examined in cells treated for 10 days with combined PFAS at 150 μM, 100 μM, 50 μM, 20 μM, and DMSO (Figure 2b–e). Exposure to PFAS significantly (*p* < 0.0001) decreased mitochondrial content in the cells treated with 150 μM PFAS (mean = 7764) compared with cells treated with DMSO (mean = 11,530), as detected by MitoTracker Red MFI. Similarly, mitochondrial membrane potential detected by TMRM signal significantly decreased in the 150 μM group (mean = 5375, *p* < 0.0001) compared with the DMSO control (mean = 11,110).

In addition, a slight increase in MitoTracker Red signal was detected in the 100 μM (mean = 13,866, *p* < 0.0001) and 50 μM (mean = 12,499, *p* < 0.05) groups. Likewise, TMRM signal slightly increased in the 100 μM (mean = 12,957, *p* < 0.0001), 50 μM (mean = 11,953, *p* < 0.05), and 20 μM (mean = 11,934, *p* < 0.05) groups. A similar phenomenon was observed in a previous study where PFAS exposure increased mitochondrial membrane potential in human endometrioid endometrial adenocarcinoma and Ishikawa cell lines.^36^

These results suggest that exposure of hiPSC-CMs to 150 μM PFAS for 10 days led to mitochondrial membrane depolarization and mitochondrial content loss, consistent with previous findings demonstrating perfluorooctanesulfonic acid (PFOS) induced downregulation of ATP synthase subunit genes in rat heart tissue.^37^

### Combined PFAS Exposure Alters Cysteine Metabolism

PFAS exposure has been associated with dysregulations to redox homeostasis in other cell types.^38^ Redox imbalance often correlates with mitochondrial dysfunction, which plays a pivotal role in cardiac defects.^39^ To evaluate the redox state in hiPSC-CM cultures treated with PFAS, we quantified reduced /oxidized glutathione (GSH/GSSG) and cysteine/cystine (Cys/CySS) using HPLC. On day 10 postexposure, both CySS and Cys levels were elevated (Figure 3), although the ratio of CySS to CySS + Cys (percentage of CySS) remained unchanged. Similarly, no significant alterations were observed in GSH, GSSG, or the GSSG percentage. The increase in the total cysteine pool suggests a shift in cysteine metabolism, potentially indicating an early adaptive mechanism to preserve redox homeostasis.

**Figure 3:**
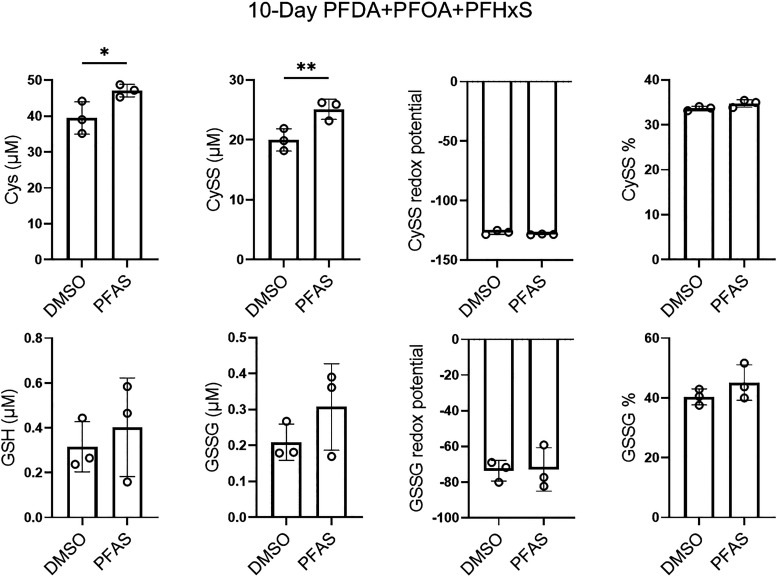
Combined PFAS exposure on hiPSC-CMs induces cysteine metabolism shift. hiPSC-CM cultures were treated with combined PFAS or DMSO for 10 days and then analyzed for redox status. Comparisons were conducted via Welch’s *t*-test. *p < 0.05; **p < 0.01. *n* = 3 cultures.

### Combined PFAS Exposure Alters Cell Composition in hiPSC-CM Differentiation Cultures

To determine whether the PFAS exposure affected cell composition, cultures were stained with markers for cardiomyocytes and fibroblasts following 14 days of treatment with combined PFAS at 150 μM, 100 μM, 50 μM, 20 μM, and DMSO. Cardiac transcription factor NKX2.5 was costained with muscle structural protein α-actinin (Figure 4), and cTnT was costained with vimentin, a fibroblast maker (Figure 5).

**Figure 4:**
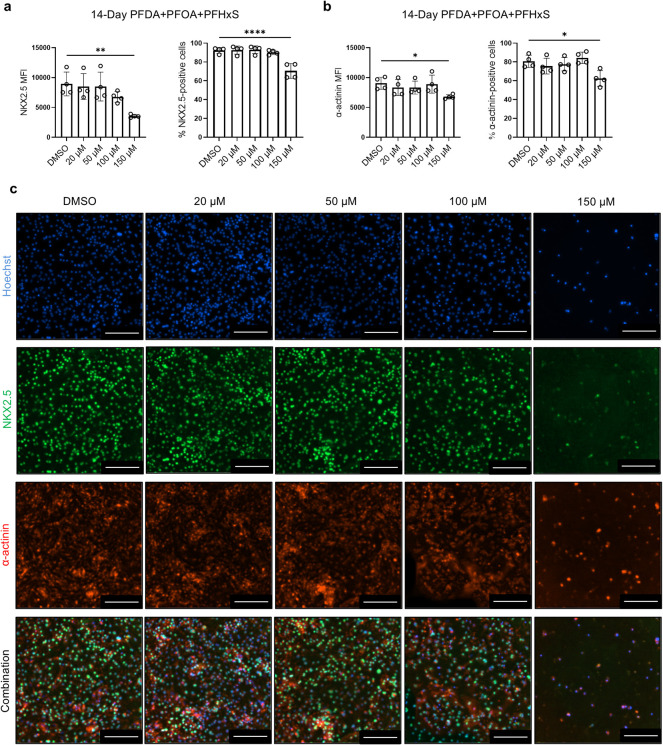
Combined PFAS exposure on hiPSC-CM reduces the expression of NKX2.5 and α-actinin. hiPSC-CM cultures were treated with combined PFAS or DMSO for 14 days and then analyzed for the expression of NKX2.5 and α-actinin by immunocytochemistry. (a) Quantification of % of NKX2.5-positive cells and mean fluorescence intensity (MFI) of NKX2.5. (b) Quantification of % of α-actinin-positive cells and MFI of α-actinin. (c) Representative images of NKX2.5 and α-actinin staining. Scale bars: 200 μm. Comparisons were conducted via one-way ANOVA test followed by Tukey’s multiple comparisons test. *p < 0.05; **p < 0.01; ****p < 0.0001. *n* = 4 cultures.

**Figure 5:**
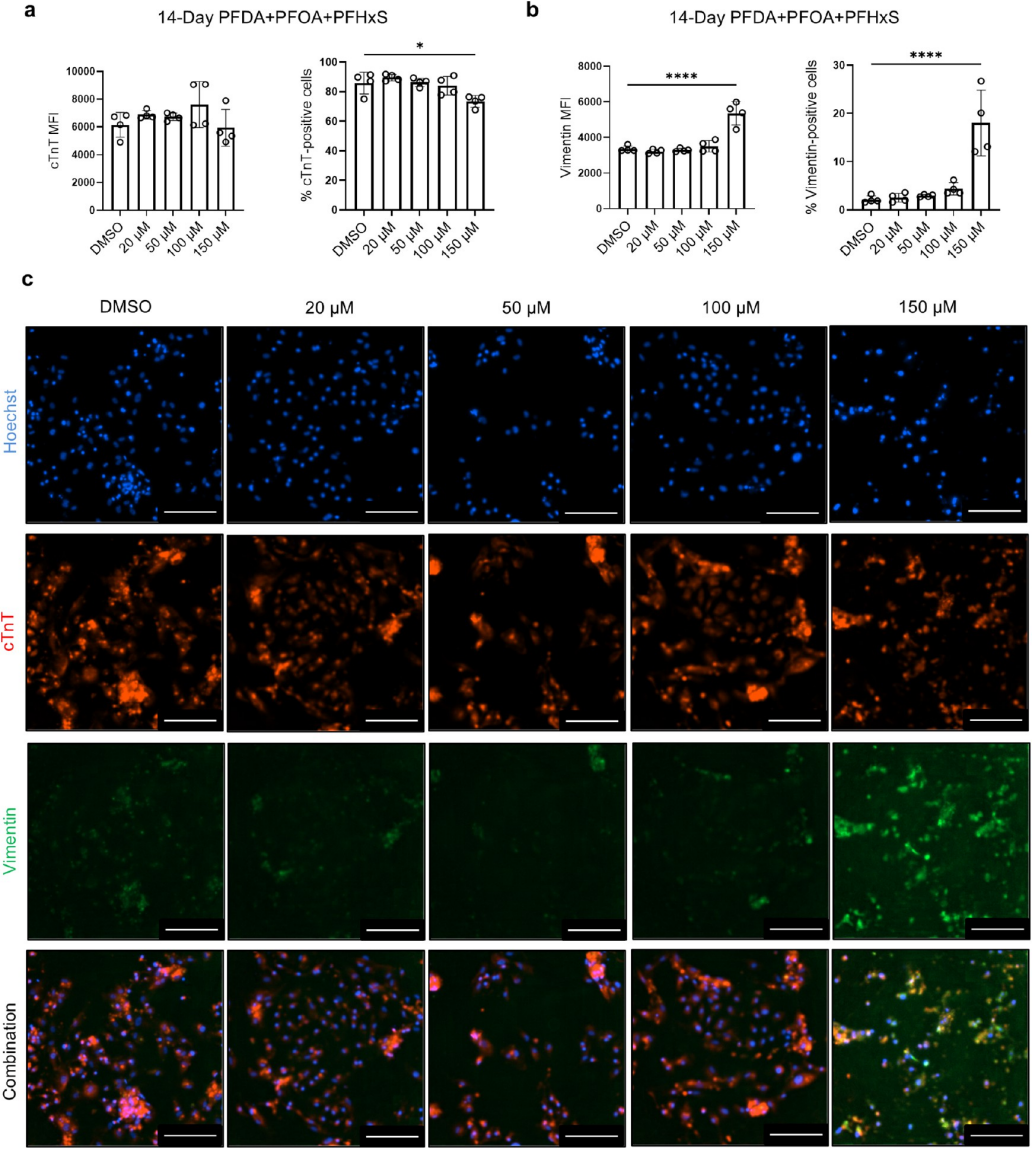
Combined PFAS exposure on hiPSC-CMs increases the expression of vimentin and decreases the expression of cTnT. hiPSC-CM cultures were treated with combined PFAS or DMSO for 14 days and then analyzed for the expression of cTnT and vimentin by immunocytochemistry. (a) Quantification of % of cTnT-positive cells and mean fluorescence intensity (MFI) of cTnT. (b) Quantification of % of vimentin-positive cells and MFI of vimentin. (c) Representative images of cTnT and vimentin staining. Scale bars: 200 μm. Comparisons were conducted via one-way ANOVA test followed by Tukey’s multiple comparisons test. *p < 0.05; ****p < 0.0001. *n* = 4 cultures.

Immunocytochemistry analysis revealed a significant reduction in NKX2.5 MFI in the 150 μM PFAS group (mean = 3561, *p* < 0.01) compared with the DMSO control (mean = 8937) (Figure 4a). Similarly, the percentage of NKX2.5-positive cells was significantly decreased in the 150 μM group (mean = 70.6%, *p* < 0.0001) relative to control (mean = 92.3%). Consistent with prior reports of α-actinin downregulation in ESC-derived cardiomyocytes exposed to PFOS,^13,36^ a significant reduction was observed in α-actinin MFI (mean = 6792, *p* < 0.05) and the percentage of α-actinin-positive cells (mean = 62.4%, *p* < 0.05) in the 150 μM group, compared with the DMSO control (mean MFI = 9046; mean % positive cells = 80.7%) (Figure 4b).

While cTnT MFI did not show significant differences between PFAS-treated and control groups, the percentage of cTnT-positive cells was significantly reduced in the 150 μM group (mean = 73.4%, *p* < 0.05) compared with control (mean = 85.8%) (Figure 5a). In contrast, vimentin MFI was significantly increased in the 150 μM group (mean = 5357, *p* < 0.0001) relative to control (mean = 3357) (Figure 5b). Similarly, the percentage of vimentin-positive cells was markedly elevated in the 150 μM group (mean = 18%, *p* < 0.0001) compared with control (mean = 2.2%). These findings are consistent with previous reports of elevated vimentin expression in human rhabdomyosarcoma cells exposed to PFOA,^40^ and in colorectal cancer cell lines following PFOS or PFOA treatment.^34^

Collectively, these results indicate that combined PFAS at 150 μM disrupted cardiomyocyte population in differentiation cultures by reducing cardiac marker expression while promoting fibroblast marker expression.

### Combined PFAS Exposure Alters the Proteome of hiPSC-CMs

To further investigate the molecular changes induced by PFAS, we performed the proteomics analysis comparing hiPSC-CM cultures treated with combined PFAS at 100 μM for 14 days with those treated with DMSO. The concentration of 100 μM was selected since cultures treated with PFAS at this concentration remained viable without changes in the proportion of cardiomyocytes compared with DMSO-treated cells. In total, 8062 proteins were quantified across both experimental conditions (Figure 6a). Pearson correlation analysis demonstrated high reproducibility among biological triplicates within each condition (Pearson correlation coefficient = 0.96 to 1; Figure 6b). Of these, 905 proteins were differentially abundant [ | log2­(fold change) | ≥ 1, *p*-value < 0.05], with 436 proteins exhibiting decreased abundance and 469 showing increased abundance following the PFAS exposure (Figure 6c).

**Figure 6:**
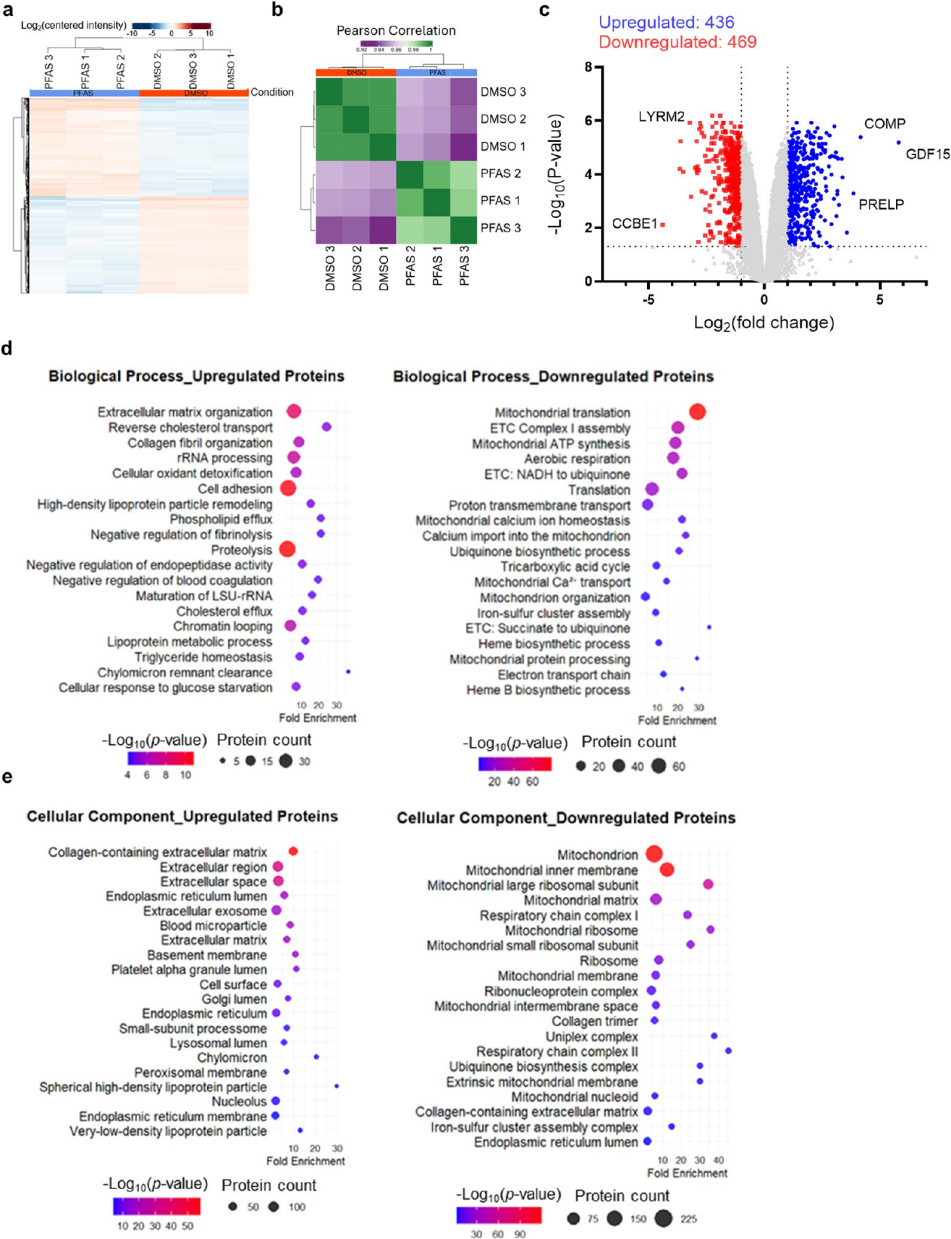

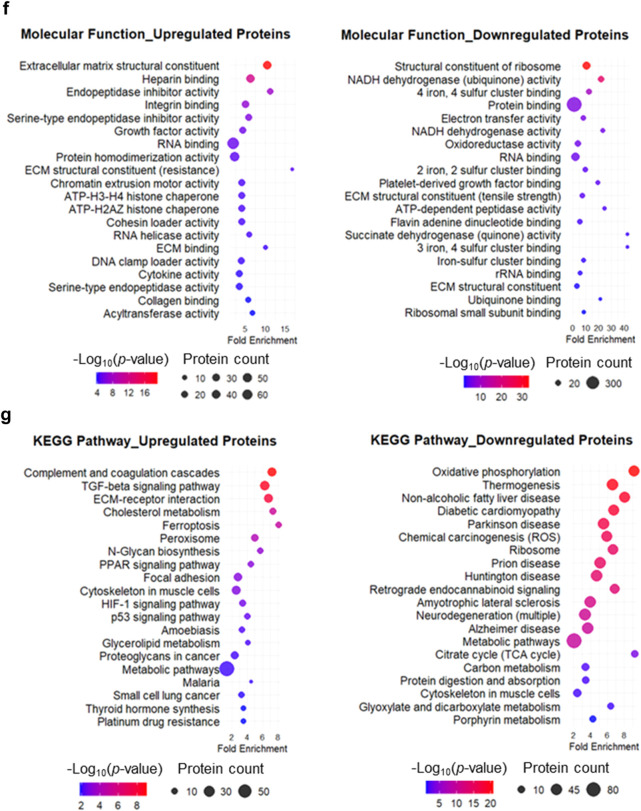
Combined PFAS exposure significantly alters the proteome of hiPSC-CMs. hiPSC-CM cultures were treated with combined PFAS at 100 μM or DMSO for 14 days and then analyzed for proteomic profiles. (a) The hierarchical clustering analysis of 8062 proteins between combined PFAS exposure and DMSO control. (b) Pearson’s correlation coefficient shows reproducibility of the biological triplicates of cell lysate samples. (c) The volcano plots illustrate upregulated (depicted in blue color) and downregulated (depicted in red color) proteins based on | log_2_(fold change) | ≥ 1 and *p*-value < 0.05 in the 14 day PFAS exposure cell samples compared with the DMSO control. GO enrichment analysis for biological process (d), cellular component (e) and molecular function (f) GO terms, and KEGG pathways (g), based on upregulated and downregulated proteins (PFAS vs DMSO). *n* = 3 cultures.

The top upregulated proteins included cartilage oligomeric matrix protein (COMP), growth/differentiation factor 15 (GDF15), prolargin (PRELP), and adenine nucleotide translocase lysine *N*-methyltransferase (ANTKMT) (Table S2). COMP is a pentameric extracellular matrix (ECM) glycoprotein that facilitates cell adhesion and can interact with transforming growth factor-beta (TGF-β), modulating its activity.^41–43^ This interaction is consistent with the observed upregulation of GDF15, a member of the TGF-β superfamily. PRELP, an ECM protein belonging to the small leucine-rich proteoglycan family, has been associated with cardiac fibrosis.^44^

Intelectin-1 (ITLN1) was among the most significantly downregulated proteins following the PFAS exposure (Table S3). ITLN1 is a secreted lectin primarily expressed in visceral adipose tissue and mesothelial cells, including those in the heart.^45^ In addition, ITLN1 can decrease ovarian cancer invasion and induce metabolic shift.^46^ In gastric cancer cells, ITLN1 increased the expression of hepatocyte nuclear factor 4 alpha, a transcription factor that suppresses the nuclear translocation and transcriptional activity of β-catenin,^45^ a central component of the canonical Wnt signaling pathway which is crucial for cell proliferation and differentiation.^47^ LYR motif-containing protein 2 (LYRM2) was also among the downregulated proteins (Table S3). LYRM2 is a mitochondrial matrix protein that supports NADH oxidation and ATP production.^48^ Furthermore, collagen and calcium binding EGF domains 1 (CCBE1), which has been linked to tumor metastasis,^49,50^ was also significantly downregulated (Table S3).

We further performed GO enrichment analysis using the upregulated and downregulated proteins (Figure 6d–f and Tables S4 and S5). Among upregulated proteins, the most significantly enriched biological process (BP) GO term was ECM organization (Figures 6d, S1a and Table S4), driven by the increased abundance of periostin (POSTN), epidermal growth factor-like protein 6 (EGFL6), and fibulin-like protein. Collagen fibril organization was also enriched and associated with the upregulation of lysyl oxidase-like 2 (LOXL2), lysyl oxidase-like 4 (LOXL4) and alpha-2-antiplasmin (A2AP). Enhanced cell adhesion process was indicated by upregulation of related proteins such as laminin subunit alpha 1 (LAMA1) and laminin subunit gamma 2 (LAMC2). In addition to ECM-related GO terms, cholesterol efflux, high-density lipoprotein particle remodeling, and cellular oxidant detoxification (Figure 6d) were also among the top GO terms enriched for upregulated proteins. Key proteins related to the GO terms of cholesterol efflux and high-density lipoprotein particle remodeling included apolipoprotein family (APOC2, APOC3, APOA1, APOA2, and APOA4) and hepatic lipase (LIPG). The upregulation of 12 proteins involved in cellular oxidant detoxification further indicated the activation of oxidative stress defense mechanisms, consistent with the redox analysis findings. The proteomics data also revealed enrichment of canonical NRF2–ARE (antioxidant response element) target proteins, including heme oxygenase-1 (HO-1) (log_2_[fold change] = 1.76; *p* < 0.001), NAD­(P)H quinone dehydrogenase 1 (NQO1) (log_2_[fold change] = 1,43; *p* < 0.01), and solute carrier family 7 member 11 (SLC7A11) (log_2_[fold change] = 2.5; *p* < 0.0001). These results suggest that the enriched antioxidant defense pathways might play a role as a compensatory response to PFAS stress.

Conversely, GO terms associated with downregulated proteins were predominantly related to mitochondrial function and energy metabolism. These GO terms included biological process of mitochondrial translation (mitochondrial ribosomal protein gene family), mitochondrial respiratory chain complex I assembly, and mitochondrial ATP synthesis (Figure 6d), and cellular component (CC) of mitochondrion (Figures 6e and S1b), collectively suggesting a suppression of mitochondrial function.

GO term molecular function (MF) analysis further emphasized the ECM remodeling phenotype. Among upregulated proteins, ECM constituent was identified as a top enriched MF GO term, highlighting changes in ECM (Figure 6f). Another top enriched MF GO term was integrin binding, reflecting upregulation of ligands to integrins, which are a family of transmembrane cell surface proteins and mediate cell–cell and cell-ECM interactions.^51^ The additional top enriched MF GO term was serine-type endopeptidase inhibitor activity. Upregulated proteins related to this GO term included plasminogen activator inhibitor-1 (PAI-1) and heparin cofactor II (HCII), which are known to be involved in fibrosis procedures in the heart.^52,53^ This result was consistent with the enriched GO BP term of negative regulation of fibrinolysis (Figure 6d).

Notably, MF GO terms also highlighted mitochondrial dysfunction, especially electron transport (Figure 6f). Enriched MF GO term of NADH dehydrogenase (ubiquinone) activity (22 proteins) was consistent with the BP GO terms mitochondrial respiratory chain complex I assembly and proton motive force-driven mitochondrial ATP synthesis (Figure 6d). Moreover, the enrichment of “3 iron, 4 sulfur cluster binding” MF GO term among downregulated proteins further implicated alterations in components essential for mitochondrial electron transfer.

Using the upregulated and downregulated proteins, we also performed KEGG pathway analysis (Figures 6g and S2, Tables S6 and S7). The analysis revealed enriched pathways included ECM-receptor interaction and TGF-β signaling pathways, known for its role in myocardial fibrosis.^54^ Key proteins such as fibronectin 1 (FN1), laminin subunits (LAMA1 and LAMC2), and transforming growth factor beta 3 (TGFB3) indicated fibrosis and activation of TGF-β/SMAD signaling pathway.^55–57^ Consistent with ECM-related alterations, the focal adhesion pathway was also enriched. Several cholesterol-related pathways were among the top enriched pathways, which was consistent with the BP and MF GO analysis, indicating increased cholesterol clearance. Enriched peroxisome and PPAR signaling pathway suggested a shift in lipid metabolism within cardiomyocytes.^58^ Ferroptosis, a pathway associated with regulated cell death, was also among the top enriched pathways, characterized by the upregulation of iron-binding and antioxidant proteins including transferrin, ferritin, and HO-1.

Among the KEGG pathways associated with downregulated proteins, the oxidative phosphorylation pathway (Figure S2a) was the top enriched pathway, consistent with BP GO terms of mitochondrial respiratory chain complex I assembly, and proton motive force-driven mitochondrial ATP synthesis. Pathways reflecting systemic consequences of mitochondrial energy loss, such as tricarboxylic acid (TCA) cycle (Figure S2b) was also enriched, which was associated with thermogenesis (hsa04714; 39 proteins) and nonalcoholic fatty liver disease. Several disease-specific pathways were enriched, including diabetic cardiomyopathy, Parkinson’s disease, Huntington’s disease, and Alzheimer’s disease. Their enrichment herein was predominantly driven by the common downregulation of mitochondrial complex components.^59–62^ Another highly significant pathway was chemical carcinogenesis (reactive oxygen species) which was also driven by depletion of mitochondrial electron transport proteins. Additionally, the ribosome pathway was enriched, consistent with the enriched CC GO term of mitochondrial large ribosomal subunit, suggesting impacts on mitochondrial protein synthesis machinery.

We also conducted a pathway-level visualization using KEGG Mapper to overlay differentially abundant proteins onto pathway diagrams. As shown in the ECM–receptor interaction map (Figure S3), the PFAS exposure was associated with increased ECM-related proteins, including FN1, LAMA1, and LAMA2 as well as the matricellular component POSTN, with concurrent elevation of matrix-stabilizing/cross-linking and antifibrinolytic factors (LOXL2 and PAI-1). No prominent reduced proteins (green nodes in the map) were evident in this pathway, consistent with a net gain in matrix accrual and strengthened integrin-mediated adhesion. In the TGF-β signaling map (Figure S4), we observed increased TGFB3 together with ECM coregulators (POSTN and PAI-1), indicating activation of canonical TGF-β/SMAD profibrotic signaling. Again, we did not observe reduced proteins on the map. The complement and coagulation cascade map (Figure S5) showed higher levels of serpin inhibitors relevant to fibrinolysis— PAI-1, HCII, and A2AP—which collectively suggested matrix preservation; reduced proteins were not prominent in this pathway. Together, these KEGG Mapper overlays reinforce that PFAS mixtures caused ECM remodeling in hiPSC-CM cultures—characterized by increased matrix deposition and integrin engagement—concurrently with pro-fibrotic TGF-β signaling, antifibrinolytic shifts in coagulation cascades, and augmented cholesterol metabolic processes. Finally, the cholesterol metabolism KEGG map (Figure S6) and protein network (Figure S2c) highlighted enrichment of multiple HDL-associated apolipoproteins, consistent with enhanced lipoprotein remodeling and cholesterol handling, while no clear reduced proteins were observed.

Taken together, these results demonstrate that the PFAS exposure induced significant proteome alterations in cardiomyocytes. These alterations are characterized by the downregulation of proteins involved in metabolic pathways, alongside a pronounced decrease in mitochondrial and ribosomal proteins. Concurrently, a significant dysregulation of proteins associated with the extracellular matrix was observed, which was accompanied by an increase in proteins related to lipid metabolism. Collectively, these findings indicate a transition of cellular state from predominant energy-yielding metabolic processes toward the widespread engagement of stress-response pathways.

### Combined PFAS Exposure Impairs Mitochondrial Function in hiPSC-CMs

Given the proteomics evidence of reduced mitochondrial proteins after combined PFAS exposure, we next examined mitochondrial superoxide generation and ATP levels in hiPSC-CMs. After a 14 day exposure to 100 μM combined PFAS, mitochondrial oxidative stress was significantly increased, as indicated by elevated fluorescence signal of mitochondrial superoxide indicator MitoSOX (mean MFI = 3683 in PFAS-treated cells vs 2605 in DMSO controls, *p* < 0.01) (Figure 7a,b). In addition, ATP levels were severely decreased, with 78.4% reduction (*p* < 0.0001) in the cells treated with 100 μM combined PFAS for 14 days compared with the controls (Figure 7c).

**Figure 7:**
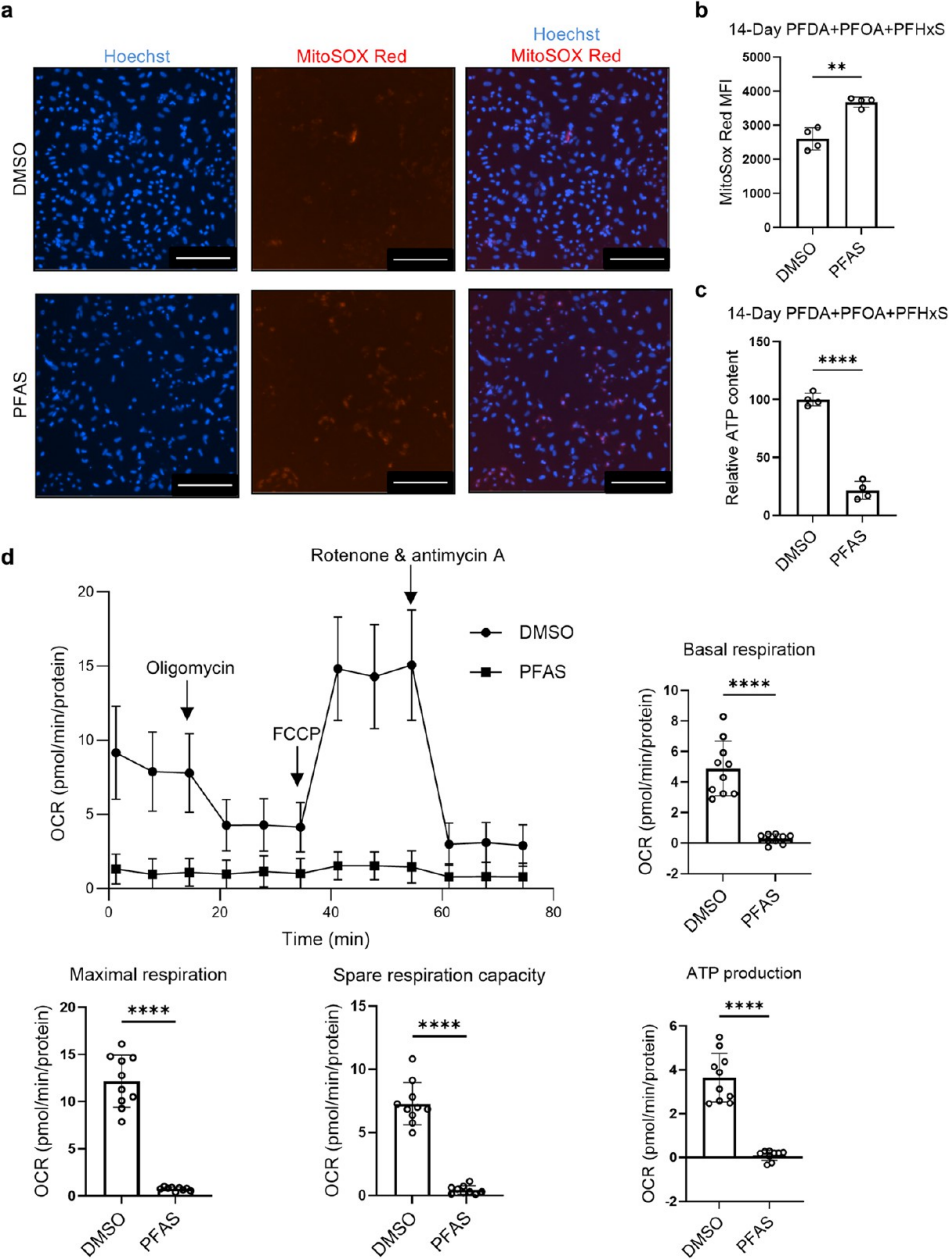
Combined PFAS exposure on hiPSC-CMs impairs mitochondrial function and reduces cellular respiration. hiPSC-CM cultures were treated with combined PFAS at 100 μM or DMSO for 14 or 18 days and then analyzed for mitochondrial oxidative stress and mitochondrial function. (a) Representative images of MitoSOX Red staining. Scale bars: 200 μm. (b) Quantification of mean fluorescence intensity (MFI) of MitoSOX Red. **p < 0.01. *n* = 4 cultures. (c) Quantification of normalized ATP content. ****p < 0.0001. *n* = 4 cultures. (d) Traces of oxygen consumption rate (OCR) and quantification of basal respiration, maximal respiration, spare respiratory capacity, and ATP production. Comparisons were conducted via Welch’s *t*-test. ****p < 0.0001. *n* = 10 (DMSO) and *n* = 9 (PFAS) technical replicates.

We next evaluated the effect of combined PFAS exposure on cellular respiration in hiPSC-CMs using Seahorse XF analysis (Mito Stress test) after the cells were treated with combined PFAS at 100 μM for 18 days. Compared with DMSO controls, the PFAS-treated cells had dramatically lower oxygen consumption in all measured parameters (Figure 7d): reduced basal respiration (mean OCR = 0.2934 vs 4.883 in DMSO controls, *p* < 0.0001), maximal respiration (mean OCR = 0.7565 vs 12.17 in DMSO controls, *p* < 0.0001), spare capacity (mean OCR = 0.4631 vs 7.282 in DMSO controls, *p* < 0.0001), and ATP production (mean OCR = 0.1044 vs 3.646 in DMSO control, *p* < 0.0001). Combined PFAS exposure also lowered basal ECAR in the hiPSC-CMs (mean ECAR = 1.464 vs 2.492 in DMSO controls, *p* < 0.01) (Figure S7).

These functional assessments demonstrate that combined PFAS exposure induced significant mitochondrial oxidative stress and profoundly impaired mitochondrial function in hiPSC-CMs. Consistently, the proteomics analyses also indicate reduced mitochondrial/oxidative phosphorylation (OXPHOS) and TCA-related proteins with concurrent activation of antioxidant programs (e.g., HO-1, NQO1, and SLC7A11) (Figure 6 and Tables S4–S7), supporting interference with metabolic pathways in the PFAS-treated condition. Therefore, the functional mitochondrial impairment aligns with proteomics findings of widespread reduction of mitochondrial respiratory chain and ATP synthesis proteins in PFAS-treated cells.

## Discussion

hiPSC-CM cultures provide a unique platform for the investigation of cellular and molecular consequence of PFAS exposure to human cardiomyocytes. Compared with hiPSC-CM cultures treated with individual PFAS compounds, a mixture of PFAS (combined PFAS exposure) resulted in synergistic cytotoxic effects.

Further characterization showed that exposure to combined PFAS disrupted mitochondrial features in hiPSC-CMs, as evidenced by alterations in mitochondrial membrane potential and content, highlighting mitochondrial vulnerability to environmental toxicants. Combined PFAS exposure also induced significant shifts in redox balance and cysteine metabolism, which may represent an adaptive response to oxidative stress within the cardiomyocytes treated with PFAS.

The synergistic cytotoxic effects observed with combined PFAS exposure in hiPSC-CMs underscore the need for comprehensive risk assessments that consider mixed exposures rather than individual compounds. Previous studies have shown the cytotoxicity effect of PFAS exposure in different cell lines. For example, dose-dependent cytotoxicity was observed in HepaG2 cells exposed to gradients of single PFAS (PFOA, perfluorononanoic acid [PFNA], and PFDA) for 3 or 24 h.^63^ However, in cardiomyocytes, a 24 h-exposure to PFOA (0.1–100 μM) did not show any significant cardiotoxicity effect.^64^ Compared with individual PFAS, combination of two PFAS compounds resulted in higher levels of cytotoxicity in HepaG2 cells.^18^ However, combined PFAS exposure has not been reported on hiPSC-CMs. In this study, we observed synergetic effects with the combined exposure of PFOA, PFDA, and PFHxS for 5 or 10 days in hiPSC-CMs.

Our results provide mechanistic insights into PFAS-induced cardiotoxicity, highlighting mitochondrial dysfunction and oxidative stress as key pathways mediating the adverse effects. One of the potential causes of cell death is mitochondrial dysfunction. Oxidative stress can trigger the release of pro-apoptosis proteins in mitochondria.^65,66^ Thus, mitochondrial content and membrane potential were tested after exposure of combined PFAS at a concentration gradient of 150 μM, 100 μM, 50 μM, 20 μM and DMSO control. After a 10-day exposure, 150 μM PFAS resulted in lower mitochondrial content and membrane potential when compared with control, which indicated mitochondrial dysfunction. A previous *in vivo* experiment showed prenatal gestational exposure to PFOS at 2 mg/kg/d caused mitochondrial swollen and downregulation of mitochondrial ATP synthetase (*ATP5E*, *ATP5I* and *ATP5O*).^37^ Another *in vivo* experiment illustrated that exposure to a mixture of PFAS at a high dose (50 μg/L) caused significant decrease in mitochondrial membrane potential and mitochondrial content.^67^ An *in vitro* study reported a similar reduction in mitochondrial membrane potential in mouse embryonic stem cell-derived cardiomyocytes exposed to 40 μM PFOS, as detected by JC-1 staining,^68^ aligning with our observations. Notably, a slight increase in mitochondrial content was observed in their research, which was also observed in our study. Since oxidative stress is one of the causes for mitochondrial failure, a redox analysis was conducted after a 10-day exposure in our study. The result showed an increase in cysteine pool, indicating alteration of cysteine metabolism after a 10-day exposure.

Combined PFAS exposure altered proteomic profiles in hiPSC-CMs related to mitochondrial function and oxidative stress, aligning with previous findings showing that one of the main adverse effects caused by PFAS exposure was mitochondrial dysfunction. Mitochondrial swelling was observed in offspring rats after prenatal exposure to PFOS.^37^ The downregulation of electron transport chain-related proteins indicated impairment of mitochondrial function and reduced ATP generation.^69^ An *in vivo* experiment on zebrafish revealed that PFOS exposure disrupted both Complex I–IV activity and related gene expression.^67^ Mitochondria are the primary source of energy and oxidative phosphorylation is the main pathway to generate ATP in mature cardiomyocytes.^69^ Thus, disruption of electron transport chain can result in energy deficit and heart failure.^70^ Alteration of contractility function^71^ and related gene expression^72,73^ have been reported in other *in vivo* PFAS exposure models. In addition, we observed enriched GO term of diabetic cardiomyopathy in hiPSC-CMs treated with PFAS. Diabetic cardiomyopathy is initially characterized by myocardial fibrosis, dysfunctional remodeling, and associated diastolic dysfunction, later by systolic dysfunction, and eventually by clinical heart failure.^74^ In line with this, proteins (HO-1, NQO1, and SLC7A11) in the ferroptosis pathway were upregulated in hiPSC-CMs treated with combined PFAS. Ferroptosis, related to oxidative and ion stress, is driven by iron-dependent phospholipid peroxidation.^75^ HO-1 is crucial in releasing iron and its upregulation contributed to iron accumulation.^76^ SLC7A11 triggers protection from oxidative stress and ferroptosis by increasing cystine uptake and glutathione biosynthesis^77^ and NQO1 is established as a superoxide reductase activity.^78^ Upregulation of these proteins in PFAS-treated hiPSC-CMs indicates activation of oxidative stress.

In addition, changes in the cellular composition of hiPSC-CM cultures upon the PFAS exposure were evident, with marked alterations in the expression of key cardiac and fibrotic markers, pointing to potential fibroblast activation which could reflect the induction of fibrotic remodeling in developmental cardiotoxicity. The PFAS exposure in hiPSC-CM cultures elicited robust enrichment of ECM remodeling pathways, notably ECM–receptor interaction, TGF-β signaling, complement and coagulation cascades, and cholesterol metabolism. The proteomics data show that multiple ECM components were enriched, including FN1, LAMA1, LAMC2, and POSTN, reflecting activation of the ECM–receptor interaction network and enhanced cell–matrix interactions. Concurrently, TGF-β signaling was activated: TGFB3 was elevated along with the collagen cross-linker LOXL2, consistent with a profibrotic stimulus. Notably, TGF-β is a master regulator of fibrosis that drives expression of ECM proteins like fibronectin,^79^ and KEGG analysis also indicates significant enrichment of the TGF-β pathway,^80^ implying that PFAS initiated the fibrotic program. Enrichment of complement and coagulation cascades further supports a remodeling milieu, with upregulated PAI-1 and A2AP, respectively—both inhibitors of fibrinolysis that reduce ECM turnover. Plasminogen activator inhibitor 1 is strongly implicated in fibrotic pathology in heart,^52^ and its enrichment in PFAS-treated cells suggests a shift toward matrix preservation. In addition, PFAS-treated cells showed dysregulation of cholesterol metabolism proteins (APOA1, APOA2, APOA4, APOC2, APOC3, and LIPG), aligning with known PFAS-associated hypercholesterolemia.^81^

Taken together, these changes suggest early cardiac fibrosis and ECM remodeling: the PFAS exposure prompted enrichment of structural matrix proteins and pro-fibrotic factors while limiting matrix degradation that suggests excessive ECM deposition. These findings are consistent with emerging *in vivo* evidence that PFAS can activate TGF-β/Smad pathways and promote fibrotic tissue remodeling.^82^ In addition, epidemiologic data have linked PFHxS levels to increased odds of liver fibrosis.^83^ Since myocardial fibrosis can disrupt electrical conductivity and relaxation—raising the risk of arrhythmias and diastolic dysfunction,^84^ future study is needed to further analyze functional consequence including contractility associated with the proteomic changes in ECM organization and metabolism.

Another major molecular change is the perturbation of proteins associated with lipid metabolism and PPAR signaling. The PPAR belongs to the nuclear receptor superfamily and mediates metabolism and fatty acid regulation, including an effect on high-density lipoprotein cholesterol levels.^85^ Significant upregulation of apolipoproteins and lipid transporters in PFAS-treated cardiomyocytes indicated enrichment of PPARα activation, fatty acid transport, and high-density lipoprotein cholesterol formation. Oral exposure to PFAS was reported to activate PPARα and PPARγ in human and rats. Proteomics analysis revealed a significant enrichment of upregulated peroxisomal membrane protein involved in the peroxisome pathway, which is regulated by PPAR and contributes to fatty acid oxidation, detoxification, and lipid metabolism. Unlike mitochondrial fatty acid β-oxidation pathway, peroxisome targets very long-chain fatty acids, which cannot be metabolized by mitochondria, and the products were shuttled to mitochondria.^86^ Given that PPAR-related pathways were enriched among upregulated proteins, the shift of fatty acid metabolism could be due to attempting of increasing energy products via fatty acid oxidation in mitochondria, providing high levels of ATP. This could be an adaptive response to the PFAS exposure since enriched BP GO term analysis revealed that mitochondrial function was damaged after the PFAS exposure in hiPSC-CMs. These results may also indicate a potential risk of lipotoxicity, since accumulation of fatty acid intermediates, especially diacylglycerol and ceramide, is harmful.^87^ Further metabolomics analysis is needed to determine detailed metabolites related to PFAS-altered lipid metabolism in cardiac cells, since abnormalities in lipid metabolism may be associated with cardiac risk in exposed populations.

Together with cellular characterization, the proteome alterations in hiPSC-CMs induced by combined PFAS suggest a multifaceted impact on human cardiomyocytes. Mitochondrial content and membrane potential markedly decreased, undermining cardiac energy supply, and contributing to eventual contractile dysfunction. At the same time, PPARα-driven metabolic reprogramming and cholesterol transport changes indicate that PFAS altered metabolism. Therefore, our results suggest that combined PFAS exposure compromises energy production thereby increasing the vulnerability of cardiomyocytes to the toxic effect of PFAS. Continued research integrating these proteomics findings with metabolism and other functional assays (e.g., contractility and electrophysiology) will be critical to further elucidate how PFAS contribute to cardiac damages. Nevertheless, the current findings on the alterations in cellular and molecular pathways in hiPSC-CMs exposed to PFAS have significant implications for understanding cardiac risk associated with these environmental contaminants.

The Seahorse findings indicate marked mitochondrial dysfunction in PFAS-treated hiPSC-CMs. Concurrent reductions in basal and FCCP-stimulated OCR, together with near-absent ATP-linked respiration and spare capacity, point to impaired electron-transport/OXPHOS capacity. These bioenergetic defects align with other imaging data: the PFAS exposure altered mitochondrial membrane potential and mitochondrial content. The mitochondrial dysfunction was also consistent with increased level of mitochondrial superoxide in PFAS-treated hiPSC-CMs. Notably, complexes I and III are major mitochondrial superoxide sites,^88^ which aligns with our proteomics data showing reduction of complex I–related proteins. The marked reduction in ATP content reflects a loss of mitochondrial bioenergetic capacity. Since OXPHOS is the principal source of cellular ATP in cardiomyocytes,^89^ decreased ATP indicates OXPHOS impairment. Prior work also supports multilevel PFAS perturbations of mitochondrial biology. For example, PFOS lowers mtDNA copy number and downregulates mitochondrial biogenesis genes (*PGC-1*α and *NRF1/2*) as well as electron transport chain (ETC) components.^90^ Consistent with our observation on diminished responses to oligomycin and FCCP in hiPSC-CMs treated with PFAS, PFOA can directly limit ETC activity.^91^ Similarly, high-dose PFOS can decrease proton-leak OCR in trophoblast cells.^90^

In addition, we observed enrichment of antioxidant and stress-response proteins (e.g., HO-1, NQO1, SLC7A11) in PFAS-exposed hiPSC-CMs, consistent with activation of the NRF2–ARE pathway and a compensatory response to oxidative stress. We note that complementary study is needed to directly evaluate NRF2 activation—assessing NRF2 nuclear translocation and induction of ARE targets—and probe fatty-acid β-oxidation capacity. Nevertheless, increased mitochondrial ROS coupled with ATP depletion likely contributed to cytotoxicity in PFAS-treated cardiomyocytes and corroborated the mitochondrial pathway alterations identified by proteomics. These data support that PFAS mixtures caused mitochondrial oxidative stress and compromised mitochondrial function.

Previous studies have examined the impact of individual PFAS compounds on cellular models, often overlooking the complex interactions that occur in mixed exposures. The significance of our findings lies in demonstrating that combined PFAS exposure can synergistically enhance cytotoxic effects, suggesting that risk assessments based solely on single compound exposures might underestimate the true health risks associated with PFAS. Although 200 μM exposure was selected to model upper-bound occupational scenarios, the persistence and placental transfer ability of PFAS suggest that lower, chronic fetal exposures can still be biologically meaningful.^5^ While 150 μM and 200 μM PFAS produced overt cytotoxicity, subcytotoxic doses (20–100 μM) also resulted in discernible changes in cellular and molecular characteristics. For example, 20–100 μM exposure increased mitochondrial membrane potential, and 100 μM caused redox adaptations, elevated mitochondrial oxidative stress, impaired mitochondrial function, and broad proteomic remodeling (including reduction of mitochondrial pathways with enrichment of extracellular matrix proteins). Therefore, PFAS mixtures can drive functional reprogramming at doses below those causing cell loss, supporting the relevance of lower-level exposure scenarios. Future study is needed to extend exposure duration and emphasize lower concentrations to better approximate maternal–fetal conditions.

In summary, this study contributes to a better understanding of the mechanisms underlying PFAS-induced cardiotoxicity, highlighting cytotoxic effect, mitochondrial defects, and altered proteins associated with mitochondrial function, metabolism, and fibrosis as critical targets of PFAS action. These insights pave the way for further investigations into the long-term cardiovascular consequences of PFAS exposure and underscore the need for regulatory frameworks that consider the cumulative impacts of multiple PFAS compounds.

## Supplementary Material



## Data Availability

The proteomics data sets were deposited in the public MassIVE database with the identifier MSV000097979.

## Notes

Supplementary data contains 7 Supplementary Tables and 7 Supplementary Figures. Table S1. Antibodies for immunocytochemistry, Table S2. Top 20 upregulated proteins after the PFAS exposure, Table S3. Top 20 downregulated proteins after the PFAS exposure, Table S4. Top 20 GO terms related to upregulated proteins, Table S5. Top 20 GO terms related to downregulated proteins, Table S6. Top 20 KEGG pathways related to upregulated proteins, Table S7. Top 20 KEGG pathways related to downregulated proteins, Figure S1. Protein networks for selected enriched GO terms in combined PFAS-treated hiPSC-CMs, Figure S2. Protein networks for selected enriched KEGG pathways in combined PFAS-treated hiPSC-CMs, Figure S3. KEGG Mapper overlay of the ECM–receptor interaction pathway in combined PFAS-treated hiPSC-CMs, Figure S4. KEGG Mapper overlay of TGF-β signaling in combined PFAS-treated hiPSC-CMs, Figure S5. KEGG Mapper overlay of complement and coagulation cascades in combined PFAS-treated hiPSC-CMs, Figure S6. KEGG Mapper overlay of cholesterol metabolism in combined PFAS-treated hiPSC-CMs, Figure S7. Combined PFAS exposure on hiPSC-CMs reduces extracellular acidification rate (ECAR) (PDF)

## Abbreviations

BP: biological process
CC: cellular component
CM: cardiomyocyte
ECAR: extracellular acidification rate
GO: Gene Ontology
hiPSC: human induced pluripotent stem cell
KEGG: Kyoto encyclopedia of genes and genomes
MF: molecular function
MFI: mean fluorescence intensity
OCR: oxygen consumption
OXPHOS: oxidative phosphorylation
PFAS: per- and polyfluoroalkyl substances
PFDA: perfluorodecanoic acid
PFHxS: perfluorohexanesulfonic acid
PFOA: perfluorooctanoic acid
