# Supplementary material for: Per- and Polyfluoroalkyl Substances Induce Cardiotoxicity and Alter Protein Profiles of Extracellular Matrix, Metabolism, and Mitochondrial Function in Human Cardiomyocytes
Source: Chem Res Toxicol. 2025 Nov 15;39(1):31–48. doi: 10.1021/acs.chemrestox.5c00267 (PMC12820961; doi:10.1021/acs.chemrestox.5c00267)
Supplement: Supplementary file 1 [file tx5c00267_si_001.pdf]

Supplementary data

Per- and polyfluoroalkyl substances induce cardiotoxicity and alter protein profiles of extracellular matrix, metabolism, and mitochondrial function in human cardiomyocytes

Wenhao Zhang [a], Zeyu Wang [b], Olivia Reid [a], Frank Harris [a], Kun Man [a], Matthew Wang [a], Stephanie Li [a], Lawrence C. Armand [a], Alicia Lane [c], Gayatri Patel [d], Victor Faundez [c], Yuhong Du [e], Ronghu Wu [b], Lou Ann Brown [a], W. Michael Caudle [f], Chunhui Xu [a, d, *]

[a] Department of Pediatrics, Emory University School of Medicine and Children's Healthcare of Atlanta, Georgia 30322, United States

[b] School of Chemistry and Biochemistry and the Petit Institute for Bioengineering and Bioscience, Georgia Institute of Technology, Atlanta, Georgia 30332, United States

[c] Department of Cell Biology, Emory University School of Medicine, Georgia 30322, United States

[d] Wallace H. Coulter Department of Biomedical Engineering, Georgia Institute of Technology and Emory University, Atlanta, Georgia 30332, United States

[e] Department of Pharmacology and Chemical Biology, Emory University School of Medicine, Atlanta, Georgia 30322, United States

[f] Gangarosa Department of Environmental Health, Rollins School of Public Health, Emory University, Atlanta, Georgia 30322, United States

[*] Address correspondence to chunhui.xu@emory.edu

Table of contents

Table S1. Antibodies for immunocytochemistry

Table S2. Top 20 upregulated proteins after the PFAS exposure

Table S3. Top 20 downregulated proteins after the PFAS exposure

Table S4. Top 20 GO terms related to upregulated proteins

Table S5. Top 20 GO terms related to downregulated proteins

Table S6. Top 20 KEGG pathways related to upregulated proteins

Table S7. Top 20 KEGG pathways related to downregulated proteins

Figure S1. Protein networks for selected enriched GO terms in combined PFAS-treated hiPSC-CMs

Figure S2. Protein networks for selected enriched KEGG pathways in combined PFAS-treated hiPSC-CMs

Figure S3. KEGG Mapper overlay of the ECM–receptor interaction pathway in combined PFAS-treated hiPSC-CMs

Figure S4. KEGG Mapper overlay of TGF-β signaling in combined PFAS-treated hiPSC-CMs

Figure S5. KEGG Mapper overlay of complement and coagulation cascades in combined PFAS-treated hiPSC-CMs

Figure S6. KEGG Mapper overlay of cholesterol metabolism in combined PFAS-treated hiPSC-CMs

Figure S7. Combined PFAS exposure on hiPSC-CMs reduces extracellular acidification rate (ECAR)

Table S1. Antibodies for immunocytochemistry

Target	Isotype	Supplier	Catalog	Dilution
NKX2.5	Rabbit IgG	Cell signaling	8792S	1:1000
α-actinin	Mouse IgG1	Sigma	A7811	1:500
Vimentin	Rabbit IgG	Abcam	92547	1:200
cTnT	Mouse IgG1	Invitrogen	2976735	1:100
Goat anti-mouse IgG1 Alexa Fluor ® 594		Invitrogen	A21125	1:1000
Goat anti-rabbit IgG (H+L) Alexa Fluor ® 488		Invitrogen	A11034	1:1000

Note: IgG = Immunoglobulin G; H+L = Heavy and Light chains. Dilutions refer to working concentrations for immunostaining. All antibodies were used as recommended by the manufacturers.

Table S2. Top 20 upregulated proteins after the PFAS exposure

Protein ID	Gene symbol	Annotation	Log_2(fold change)	*p*-value
Q99988	GDF15	growth differentiation factor 15	5.8	6.52E-06
P49747	COMP	cartilage oligomeric matrix protein	4.16	4.06E-06
P51888	PRELP	proline and arginine rich end leucine rich repeat protein	3.85	5.18E-04
Q9BQD7	ANTKMT	adenine nucleotide translocase lysine methyltransferase	3.56	1.50E-02
Q06828	FMOD	fibromodulin	3.37	1.30E-04
P05452	CLEC3B	C-type lectin domain family 3 member B	3.34	2.66E-05
Q9Y255	PRELID1	PRELI domain containing 1	3.22	1.38E-03
P04003	C4BPA	complement component 4 binding protein alpha	3.18	2.60E-05
O60687	SRPX2	sushi repeat containing protein X-linked 2	3.15	2.45E-05
P69891	HBG1	hemoglobin subunit gamma 1	3.15	4.24E-04
P02656	APOC3	apolipoprotein C3	3.14	5.61E-04
P02655	APOC2	apolipoprotein C2	3.11	3.64E-03
P02765	AHSG	alpha 2-HS glycoprotein	3.09	1.51E-05
Q9BUD6	SPON2	spondin 2	3.04	2.25E-05
Q99541	PLIN2	perilipin 2	3.02	1.65E-06
O94907	DKK1	dickkopf WNT signaling pathway inhibitor 1	3	8.25E-04
Q9NYF3	FAM53C	family with sequence similarity 53 member C	2.91	2.07E-05
Q9BQI4	CCDC3	coiled-coil domain containing 3	2.89	1.02E-04
Q9H903	MTHFD2L	methylenetetrahydrofolate dehydrogenase (NADP+ dependent 2 like	2.89	7.28E-03
Q8N8Q8	COX18	(cytochrome c oxidase assembly factor COX18	2.84	4.98E-02

Note: Data derived from proteomic analysis of hiPSC-CMs exposed to 100 µM combined PFAS for 14 days compared with DMSO control. Log_2(fold change) and *p* values were calculated using empirical Bayes statistics. Proteins were considered upregulated if they met the threshold of log_2(fold change) ≥ 1 (at least 2-fold increase) and *p*-value < 0.05.

Table S3. Top 20 downregulated proteins after the PFAS exposure

Protein ID	Gene symbol	Annotation	Log_2(fold change)	*p*-value
Q6UXH8	CCBE1	collagen and calcium binding EGF domains 1	-4.41	7.84E-03
Q8WWA0	ITLN1	intelectin 1	-3.66	6.28E-05
P02461	COL3A1	collagen type III alpha 1 chain	-3.63	5.85E-06
Q99757	TXN2	thioredoxin 2	-3.51	8.11E-05
Q9NU23	LYRM2	LYR motif containing 2	-3.23	1.19E-06
Q9BQ48	MRPL34	mitochondrial ribosomal protein L34	-3.17	6.96E-04
Q6ZTI6	RFLNA	refilin A	-2.99	5.61E-05
P02452	COL1A1	collagen type I alpha 1 chain	-2.93	5.91E-06
Q7Z6M4	MTERF4	mitochondrial transcription termination factor 4	-2.93	7.14E-05
Q6P161	MRPL54	mitochondrial ribosomal protein L54	-2.91	8.86E-05
Q8TCC3	MRPL30	mitochondrial ribosomal protein L30	-2.89	5.26E-05
Q6ZTN6	ANKRD13D	ankyrin repeat domain 13D	-2.85	3.39E-02
P35712	SOX6	SRY-box transcription factor 6	-2.84	7.13E-05
P08123	COL1A2	collagen type I alpha 2 chain	-2.81	2.08E-06
Q96B54	ZNF428	zinc finger protein 428	-2.78	1.19E-06
Q12882	DPYD	dihydropyrimidine dehydrogenase	-2.75	1.66E-06
Q9NV29	TMEM100	transmembrane protein 100	-2.71	4.32E-04
Q9UMS0	NFU1	NFU1 iron-sulfur cluster scaffold	-2.71	2.89E-06
O95833	CLIC3	chloride intracellular channel 3	-2.61	3.89E-06
P20336	RAB3A	RAB3A, member RAS oncogene family	-2.56	1.92E-02

Note: Data derived from proteomic analysis of hiPSC-CMs exposed to 100 μM PFAS for 14 days compared with DMSO control. Log_2(fold change) and *p* values calculated using empirical Bayes statistics. Proteins were considered upregulated if they met the threshold of log_2(fold change) ≤ -1 (at least 2-fold decrease) and *p*-value < 0.05.

Table S4. Top 20 GO terms related to upregulated proteins

Category	Term	Count	*p*-value	Fold enrichment
CC	GO:0062023~collagen-containing extracellular matrix	83	1.82E-58	10.15
CC	GO:0005576~extracellular region	142	4.02E-35	3.02
CC	GO:0005615~extracellular space	132	1.45E-34	3.18
MF	GO:0005201~extracellular matrix structural constituent	28	6.77E-20	10.52
CC	GO:0005788~endoplasmic reticulum lumen	40	1.95E-19	6.13
CC	GO:0070062~extracellular exosome	112	8.70E-19	2.39
CC	GO:0072562~blood microparticle	30	1.35E-18	8.54
CC	GO:0031012~extracellular matrix	34	1.44E-18	7.13
CC	GO:0005604~basement membrane	23	7.55E-17	11.11
MF	GO:0008201~heparin binding	25	1.60E-12	6.30
CC	GO:0031093~platelet alpha granule lumen	16	7.80E-12	11.26
BP	GO:0030198~extracellular matrix organization	23	1.73E-11	6.25
CC	GO:0009986~cell surface	42	6.51E-10	3.02
CC	GO:0005796~Golgi lumen	17	7.43E-10	7.60
BP	GO:0043691~reverse cholesterol transport	9	9.91E-10	24.19
CC	GO:0005783~endoplasmic reticulum	58	3.34E-09	2.34
BP	GO:0030199~collagen fibril organization	13	2.04E-08	8.86
MF	GO:0004866~endopeptidase inhibitor activity	11	3.21E-08	11.27
BP	GO:0006364~rRNA processing	17	4.82E-08	5.71
MF	GO:0005178~integrin binding	18	1.21E-07	5.01

Note: GO = Gene Ontology; CC = Cellular Component; MF = Molecular Function; BP = Biological Process. GO enrichment was performed using Database for Annotation, Visualization, and Integrated Discovery (DAVID). Fold enrichment reflects the observed vs. expected gene count ratio. *p*-values were adjusted using Benjamini-Hochberg FDR correction.

Table S5. Top 20 GO terms related to downregulated proteins

Category	Term	Count	*p*-value	Fold enrichment
CC	GO:0005739~mitochondrion	224	5.78E-120	5.92
CC	GO:0005743~mitochondrial inner membrane	143	6.65E-119	12.61
BP	GO:0032543~mitochondrial translation	63	1.05E-79	29.44
CC	GO:0005762~mitochondrial large ribosomal subunit	43	1.77E-58	34.72
CC	GO:0005759~mitochondrial matrix	66	1.21E-34	6.74
MF	GO:0003735~structural constituent of ribosome	47	7.83E-34	10.67
BP	GO:0032981~mitochondrial respiratory chain complex I assembly	31	1.44E-31	20.03
BP	GO:0042776~proton motive force-driven mitochondrial ATP synthesis	28	1.20E-27	18.92
BP	GO:0009060~aerobic respiration	28	8.49E-27	17.83
CC	GO:0045271~respiratory chain complex I	24	2.34E-26	23.59
CC	GO:0005761~mitochondrial ribosome	19	1.30E-25	35.80
BP	GO:0006120~mitochondrial electron transport, NADH to ubiquinone	23	1.91E-24	21.96
MF	GO:0008137~NADH dehydrogenase (ubiquinone) activity	22	2.23E-23	21.84
BP	GO:0006412~translation	38	8.06E-22	7.62
CC	GO:0005763~mitochondrial small ribosomal subunit	19	1.72E-21	25.27
CC	GO:0005840~ribosome	30	2.61E-18	8.32
CC	GO:0031966~mitochondrial membrane	24	3.80E-12	6.38
MF	GO:0051539~4 iron, 4 sulfur cluster binding	14	3.58E-11	12.71
BP	GO:1902600~proton transmembrane transport	23	9.35E-11	5.74
CC	GO:1990904~ribonucleoprotein complex	26	1.89E-09	4.34

Note: GO = Gene Ontology; CC = Cellular Component; MF = Molecular Function; BP = Biological Process. GO enrichment was performed using Database for Annotation, Visualization, and Integrated Discovery (DAVID). Fold enrichment reflects the observed vs. expected gene count ratio. *p*-values were adjusted using Benjamini-Hochberg FDR correction.

Table S6. Top 20 KEGG pathways related to upregulated proteins

Pathway	Count	*p*-value	Fold enrichment
hsa04610:Complement and coagulation cascades	17	9.47E-10	7.26
hsa04350:TGF-beta signaling pathway	18	2.85E-09	6.26
hsa04512:ECM-receptor interaction	16	9.58E-09	6.75
hsa04979:Cholesterol metabolism	10	6.39E-06	7.37
hsa04216:Ferroptosis	9	1.17E-05	8.05
hsa04146:Peroxisome	11	6.31E-05	4.98
hsa00510:N-Glycan biosynthesis	8	4.59E-04	5.67
hsa03320:PPAR signaling pathway	9	8.63E-04	4.45
hsa04510:Focal adhesion	15	9.90E-04	2.78
hsa04820:Cytoskeleton in muscle cells	16	1.27E-03	2.59
hsa04066:HIF-1 signaling pathway	10	2.37E-03	3.45
hsa04115:p53 signaling pathway	8	3.61E-03	4.01
hsa05146:Amoebiasis	9	5.92E-03	3.28
hsa00561:Glycerolipid metabolism	7	7.30E-03	4.05
hsa05205:Proteoglycans in cancer	13	8.05E-03	2.39
hsa01100:Metabolic pathways	57	9.12E-03	1.37
hsa05144:Malaria	6	1.01E-02	4.51
hsa05222:Small cell lung cancer	8	1.16E-02	3.23
hsa04918:Thyroid hormone synthesis	7	1.43E-02	3.51
hsa01524:Platinum drug resistance	7	1.43E-02	3.51

Note: KEGG = Kyoto Encyclopedia of Genes and Genomes. KEGG pathway enrichment was performed using Database for Annotation, Visualization, and Integrated Discovery (DAVID). Fold enrichment = observed/expected count ratio; *p*-values calculated using hypergeometric test.

Table S7. Top 20 KEGG pathways related to downregulated proteins

Pathway	Count	*p*-value	Fold enrichment
hsa00190:Oxidative phosphorylation	32	2.00E-21	9.18
hsa04714:Thermogenesis	39	9.55E-21	6.57
hsa04932:Non-alcoholic fatty liver disease	32	1.15E-19	8.07
hsa05415:Diabetic cardiomyopathy	35	5.56E-19	6.76
hsa05012:Parkinson disease	38	1.12E-17	5.55
hsa05208:Chemical carcinogenesis - reactive oxygen species	34	1.15E-16	5.93
hsa05020:Prion disease	36	1.43E-15	5.12
hsa03010:Ribosome	29	1.67E-15	6.67
hsa05016:Huntington disease	37	7.81E-15	4.71
hsa04723:Retrograde endocannabinoid signaling	26	2.86E-14	6.91
hsa05014:Amyotrophic lateral sclerosis	37	1.75E-12	3.95
hsa05022:Pathways of neurodegeneration - multiple diseases	41	1.42E-11	3.36
hsa05010:Alzheimer disease	36	3.72E-11	3.64
hsa01100:Metabolic pathways	80	9.49E-11	2.02
hsa00020:Citrate cycle (TCA cycle)	7	8.48E-05	9.23
hsa01200:Carbon metabolism	10	2.54E-03	3.41
hsa04974:Protein digestion and absorption	9	4.87E-03	3.39
hsa04820:Cytoskeleton in muscle cells	14	5.72E-03	2.39
hsa00630:Glyoxylate and dicarboxylate metabolism	5	7.20E-03	6.38
hsa00860:Porphyrin metabolism	5	2.79E-02	4.30

Note: KEGG = Kyoto Encyclopedia of Genes and Genomes. KEGG pathway enrichment was performed using Database for Annotation, Visualization, and Integrated Discovery (DAVID). Fold enrichment = observed/expected count ratio; *p*-values calculated using hypergeometric test.

a

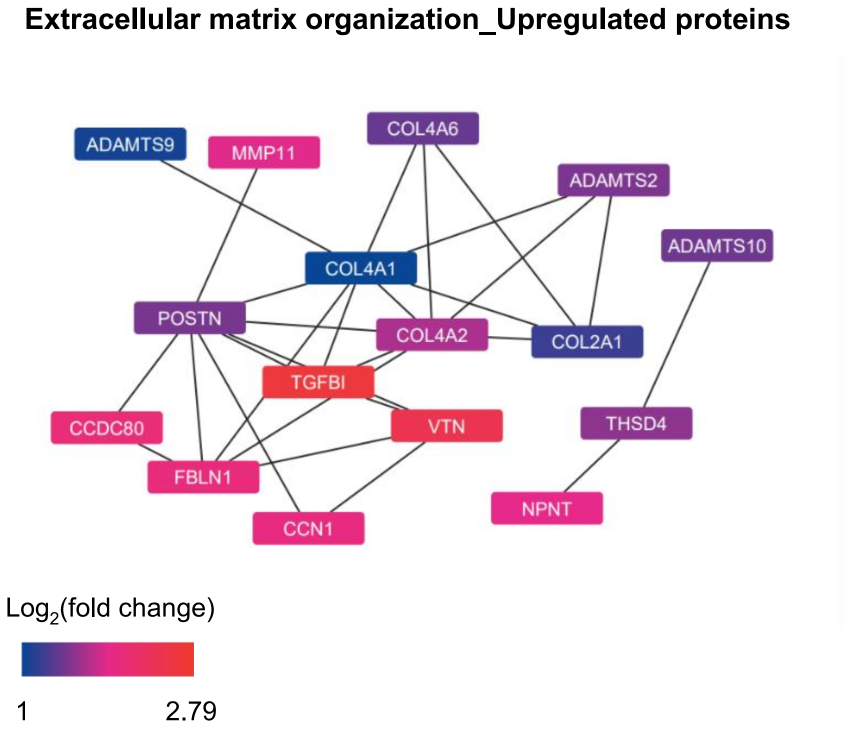

b

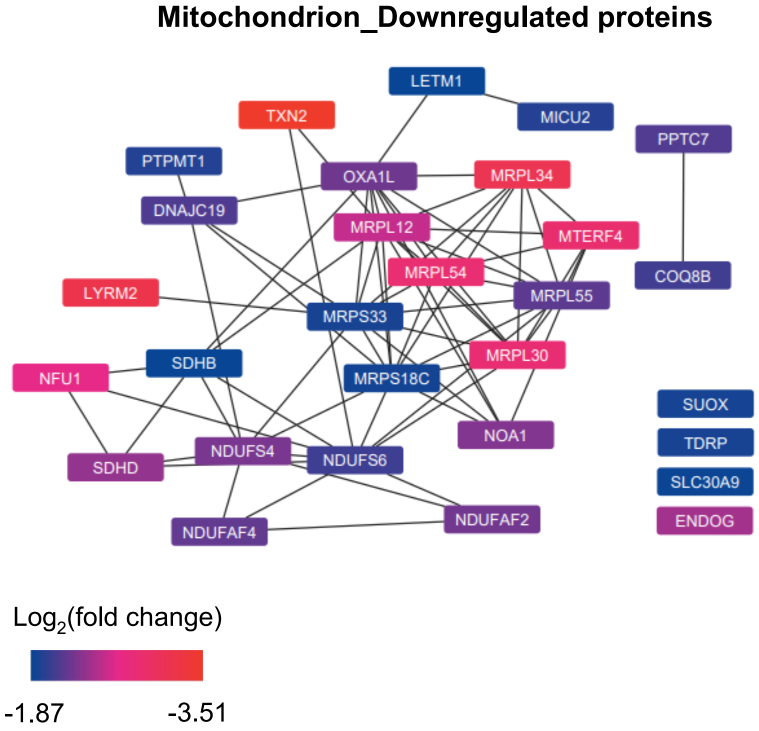

Figure S1. Protein networks for selected enriched GO terms in combined PFAS-treated hiPSC-CMs. (a) Extracellular matrix organization (n=16). (b) Mitochondrion (n=29 out of 224)

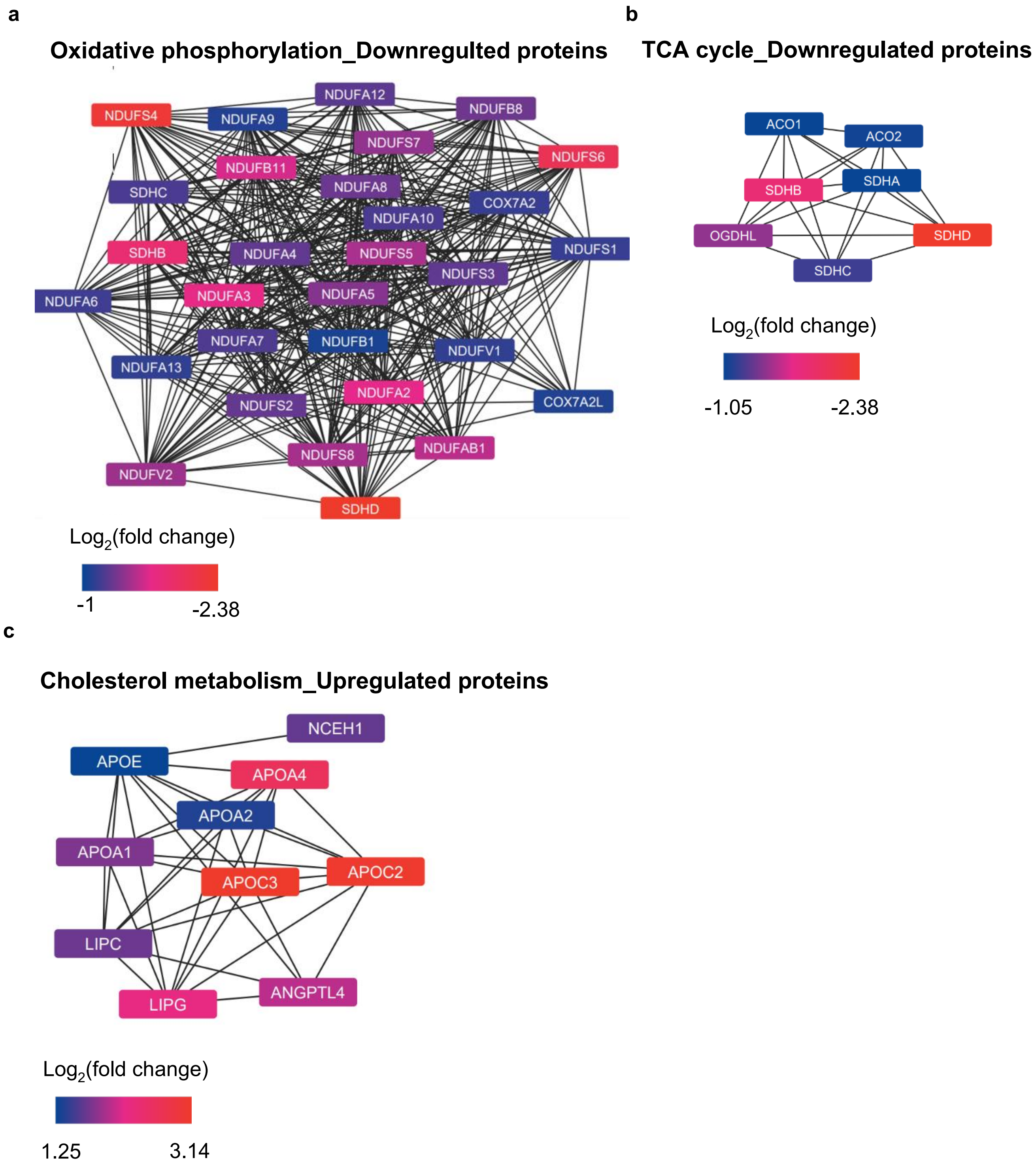

Figure S2. Protein networks for selected enriched KEGG pathways in combined PFAS-treated hiPSC-CMs. (a) Oxidative phosphorylation (n=30 out of 32). (b) TCA cycle (n=7). (c) Cholesterol metabolism (n=10).

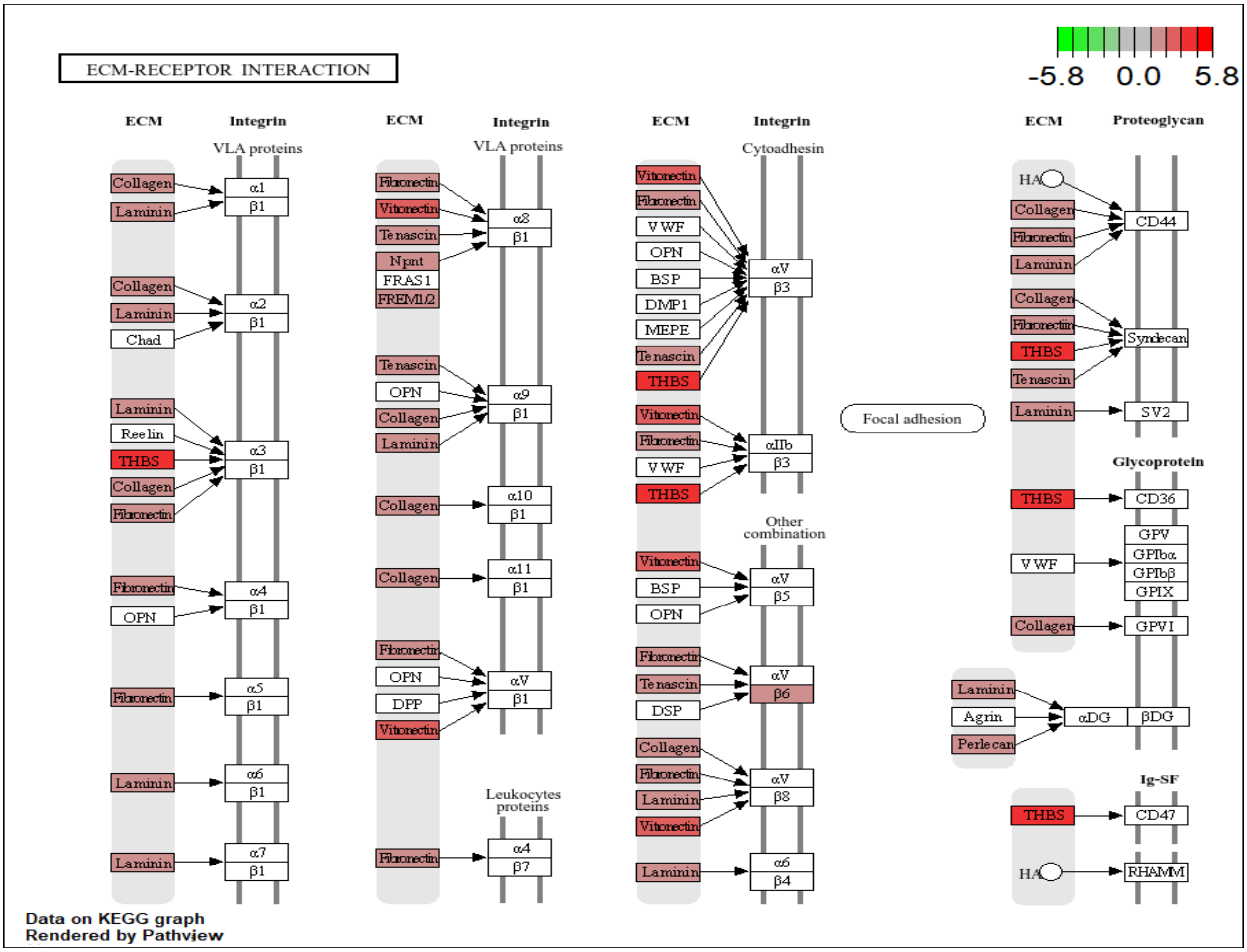

Figure S3. KEGG Mapper overlay of the ECM–receptor interaction pathway in combined PFAS-treated hiPSC-CMs. Proteins detected by proteomics and mapping to this pathway are shown on the canonical KEGG diagram; red nodes indicate upregulated proteins, and green nodes indicate downregulated proteins relative to DMSO control.

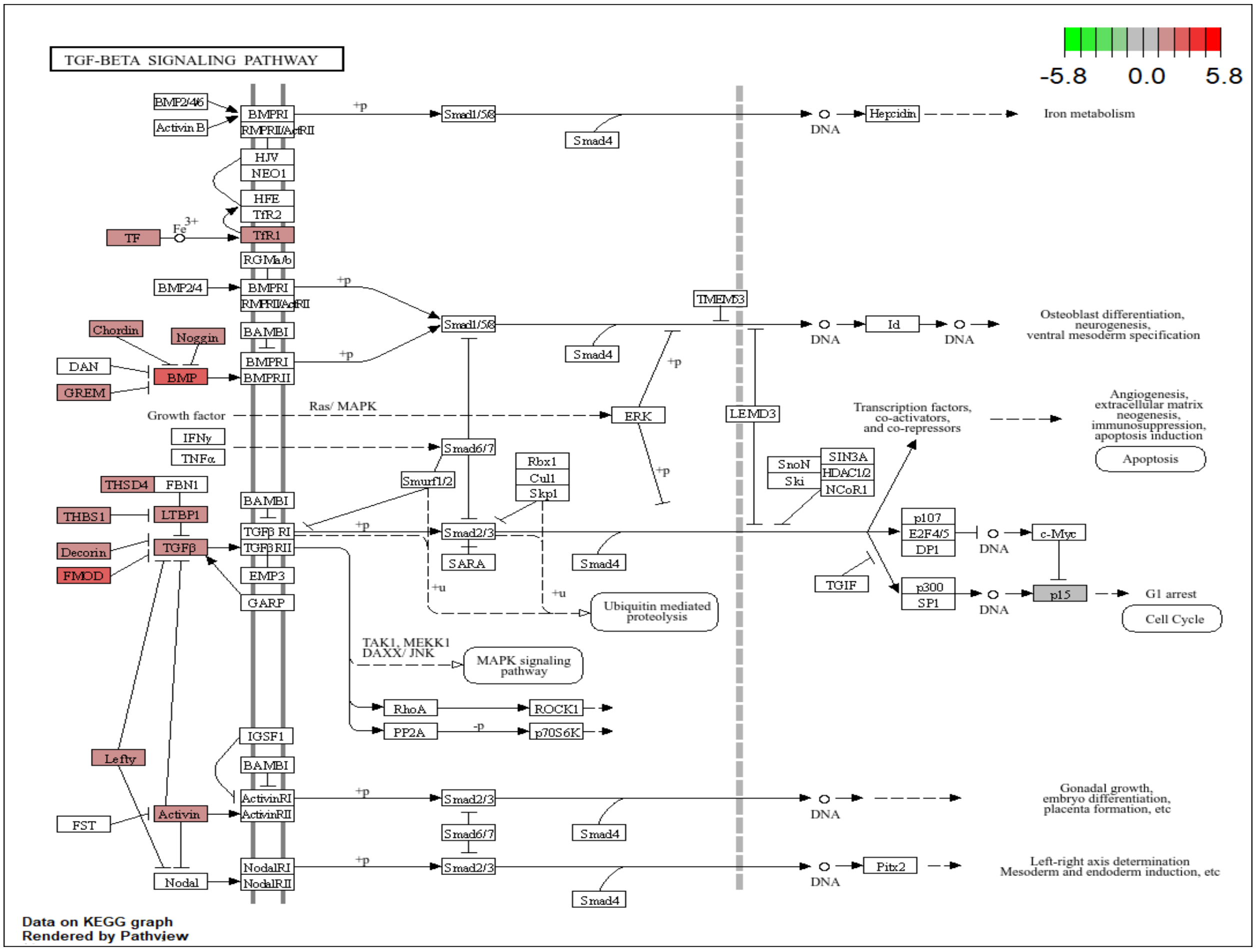

Figure S4. KEGG Mapper overlay of TGF-β signaling in combined PFAS-treated hiPSC-CMs. Proteins detected by proteomics and mapping to this pathway are shown on the canonical KEGG diagram; red nodes indicate upregulated proteins, and green nodes indicate downregulated proteins relative to DMSO control.

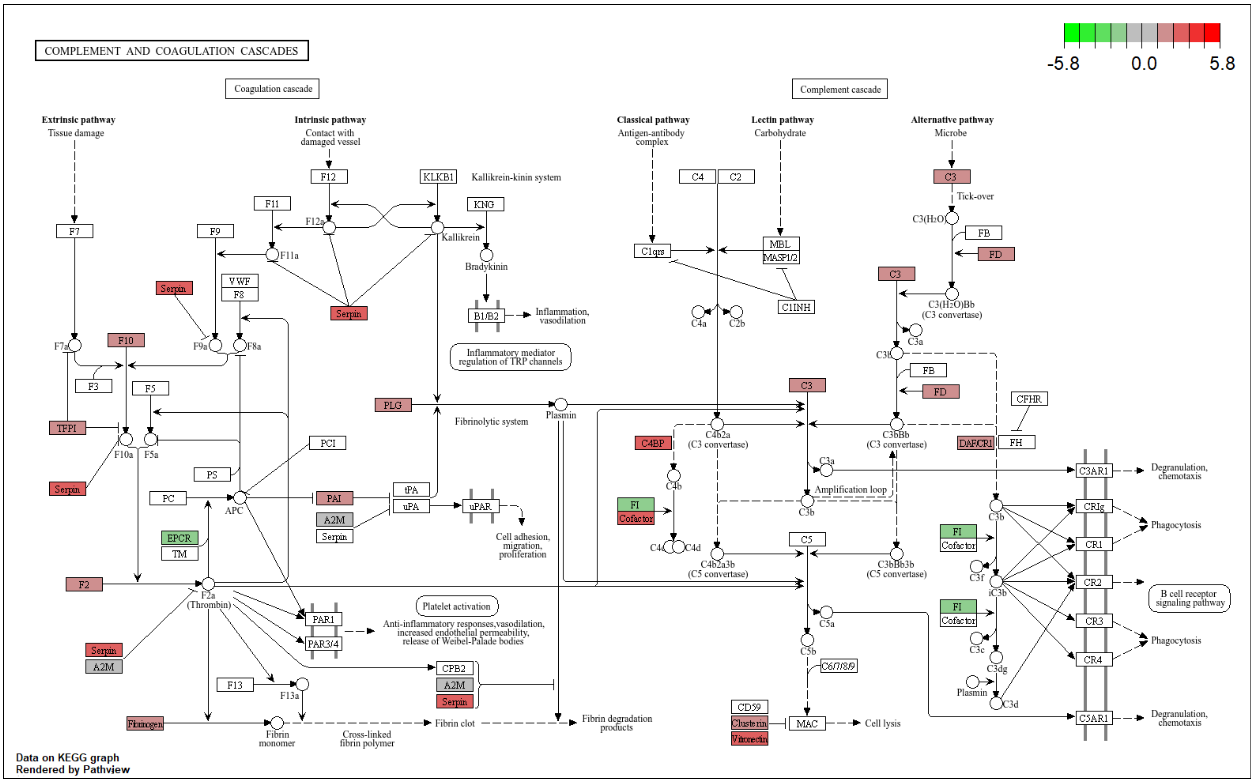

Figure S5. KEGG Mapper overlay of complement and coagulation cascades in combined PFAS-treated hiPSC-CMs. Proteins detected by proteomics and mapping to this pathway are shown on the canonical KEGG diagram; red nodes indicate upregulated proteins, and green nodes indicate downregulated proteins relative to DMSO control.

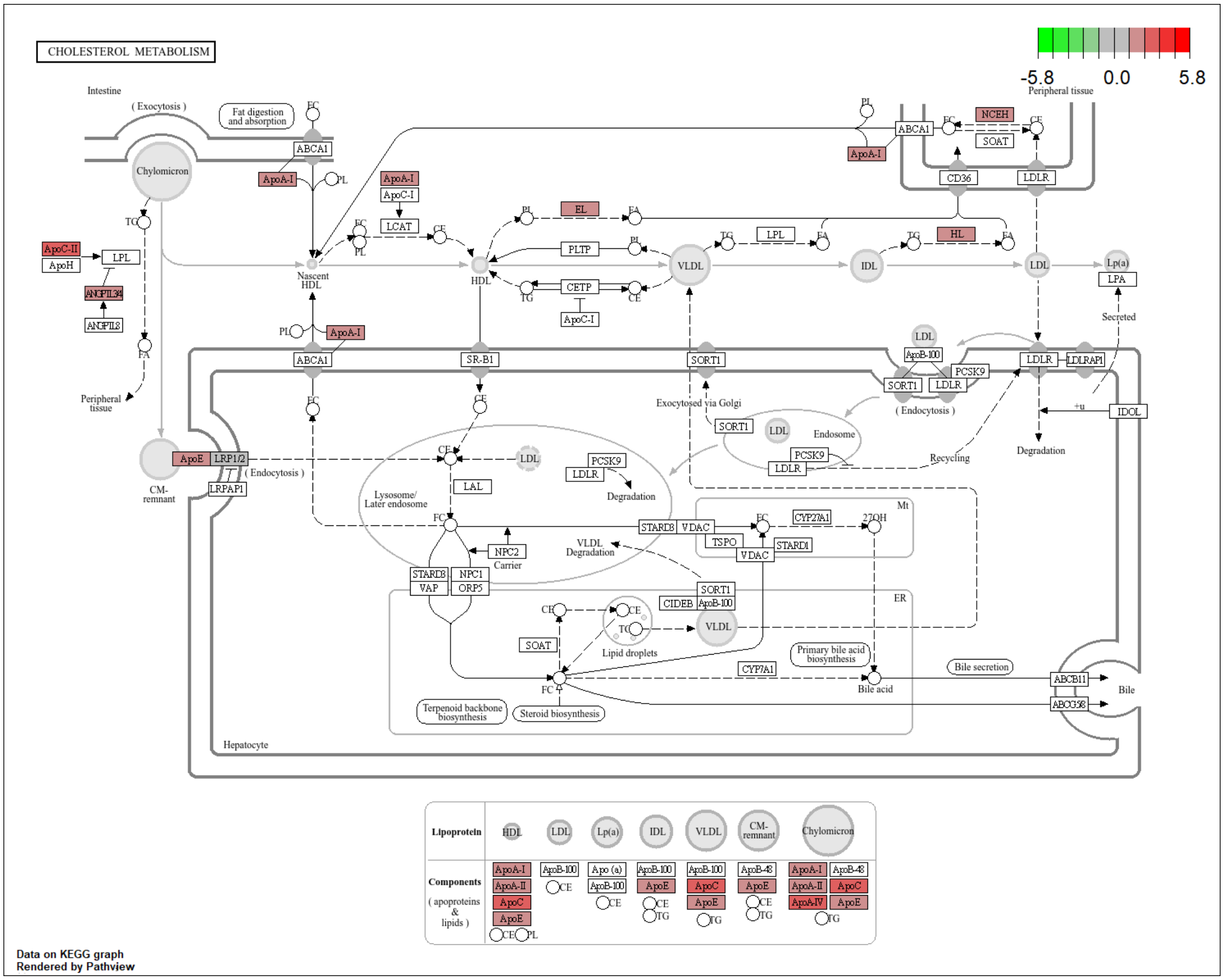

Figure S6. KEGG Mapper overlay of cholesterol metabolism in combined PFAS-treated hiPSC-CMs. Proteins detected by proteomics and mapping to this pathway are shown on the canonical KEGG diagram; red nodes indicate upregulated proteins, and green nodes indicate downregulated proteins relative to DMSO control.

a

b

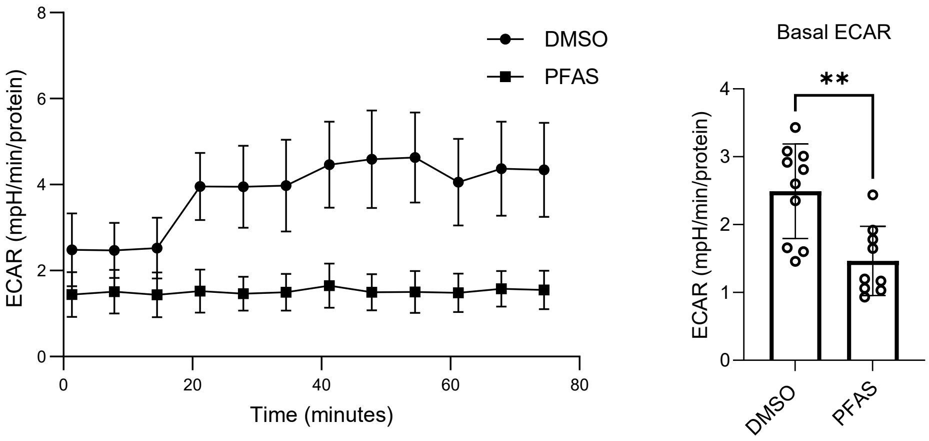

Figure S7. Combined PFAS exposure on hiPSC-CMs reduces extracellular acidification rate (ECAR). hiPSC-CM cultures were treated with combined PFAS at 100 μM or DMSO for 18 days and then analyzed. (a) Traces of ECAR. (b) Quantification of basal ECAR. Comparisons were conducted via Welch's t test. $**p < 0.01$. n=10 (DMSO) and n = 9 (PFAS) technical replicates.
